# The Spatial Redox–Metalloptosis Axis in Liver Disease: A Hypothesis on Regional Susceptibility to Ferroptosis and Cuproptosis

**DOI:** 10.3390/antiox15091053

**Published:** 2026-08-23

**Authors:** Zhaomin Dong, Maoshen Gong, Guangji Wang, Hong Wang

**Affiliations:** 1 State Key Laboratory of Natural Medicines, China Pharmaceutical University, Nanjing 210009, China; 3121070243@cpu.edu.cn (Z.D.); 3324071809@cpu.edu.cn (M.G.); gjwang@cpu.edu.cn (G.W.); 2 Key Laboratory of Drug Metabolism and Pharmacokinetics, China Pharmaceutical University, Nanjing 210009, China; 3 Jiangsu Provincial Key Laboratory of Targetome and Innovative Drugs, Institute of Innovative Drug Discovery and Development, China Pharmaceutical University, Nanjing 210009, China

**Keywords:** liver zonation, ferroptosis, cuproptosis, spatial biology, metal metabolism, oxidative stress, ceruloplasmin

## Abstract

The pathogenesis and progression of liver diseases are characterized by marked zonal heterogeneity, yet conventional research paradigms have long overlooked this intrinsic spatial logic. Ferroptosis and cuproptosis have been widely implicated in liver disease; however, their precise intralobular distribution and zonal susceptibility patterns remain poorly defined. We present a narrative synthesis of the literature on the spatial zonation of hepatic metabolism, redox homeostasis, and metal handling, and assess their potential roles as determinants of region-specific cell death vulnerability. We propose the novel “spatial redox–metalloptosis axis” hypothesis. The pericentral zone (Zone 3), characterized by hypoxia, high cytochrome P450 activity, and a redox environment that may favor lipid peroxidation under specific pathological conditions, is hypothesized to form a ferroptosis-susceptible niche under metabolic stress. Conversely, the periportal zone (Zone 1), characterized by active copper handling and oxidative phosphorylation-dependent metabolism, is hypothesized to be preferentially vulnerable to cuproptosis (proposed hypothesis; direct zone-resolved evidence of cuproptosis execution in Zone 1 is currently absent). Ceruloplasmin is proposed as a candidate molecular link between copper and iron metabolism. We further identify shared molecular hubs and a hypothesized spatial redox–metalloptosis axis linking these two regulated cell death modalities, while direct biological crosstalk remains to be demonstrated. We also highlight critical technological, mechanistic, and translational gaps. This review aims to shift liver disease research from viewing the liver as a homogeneous organ to a functionally compartmentalized zoned ecosystem, providing a testable theoretical framework for deciphering region-specific liver injury and developing spatially informed therapeutic strategies.

## 1. Introduction

The liver, the largest parenchymal organ in the human body, functions as a highly compartmentalized metabolic factory rather than a homogeneous entity. Long-standing clinical observations have long puzzled researchers: despite the highly ordered structure of the hepatic lobule and its well-defined blood flow gradients, liver injury caused by various etiologies often has a predilection for specific lobular zones. For instance, why does alcohol, a systemically ingested substance, consistently and primarily damage the pericentral region [1]? Why does copper accumulation in Wilson’s disease preferentially injure the periportal zone [2]? This zonation-specific vulnerability suggests that the liver’s susceptibility to injury is not uniformly distributed but is governed by an intrinsic spatial logic. However, in the current era of flourishing high-throughput omics and precision medicine, this logic has been largely overlooked [3]. This limitation in the research paradigm may explain why some redox-targeted therapies (e.g., free radical scavengers and ferroptosis inhibitors) that show significant efficacy in preclinical models fail to translate into expected benefits in clinical trials [4,5]. The core argument of this review is that the spatial architecture of the liver is the key to resolving this translational conundrum, and understanding where injury occurs is as biologically and clinically critical as deciphering how it occurs.

For decades, studies in liver diseases have been plagued by profound “zonal blindness”. Traditional pathological diagnosis and therapeutic strategies often treat the liver as a homogeneous entity. Bulk omics approaches (including transcriptomics, proteomics, and metabolomics), which typically rely on whole-liver homogenates, treat the hepatic lobule as a uniform mixture of cells rather than a structured ecosystem, thereby averaging out information on spatial heterogeneity [6,7]. Although useful for identifying global pathways, these approaches obscure the significant differences in metabolism, redox state, and metal homeostasis among different zones within the hepatic lobule [8]. For example, hepatocellular carcinoma (HCC) predominantly originates in the pericentral zone (Zone 3) rather than the periportal zone (Zone 1), even though Zone 1 has greater proliferative potential [9]. In patients with metabolic dysfunction-associated steatotic liver disease (MASLD), abnormal iron deposition exhibits a clear zonal preference, while evidence for zonal copper deposition in MASLD remains limited [10]. In particular, when emerging forms of cell death, such as ferroptosis and cuproptosis, are investigated, neglecting spatial heterogeneity may lead to misinterpretation of pathogenic mechanisms. For instance, ferroptosis in liver fibrosis can both induce the death of activated hepatic stellate cells (HSCs) to inhibit fibrosis and potentially activate profibrotic phenotypes, leading to hepatocyte death, a duality that may stem from differences in the zonal microenvironment [11]. Without accounting for spatial origin, such complex effects would be difficult to decipher. Amid the burgeoning research on ferroptosis and cuproptosis, although hundreds of studies have linked these cell death modalities to liver diseases, very few have reported the specific zones in which they occur [12,13]. It is a critical omission given the exquisitely refined functional zonation of the hepatic lobule. While several excellent reviews have comprehensively summarized the general roles of iron and copper metabolism in liver diseases [14] and the role of macrophages in hepatic iron homeostasis [15], the spatial dimension of metal homeostasis and its direct intersection with regulated cell death (RCD) remains largely overlooked. This review distinguishes itself by proposing the “spatial redox–metalloptosis axis” hypothesis. Here, metalloptosis is used as an umbrella conceptual term encompassing ferroptosis and cuproptosis—two metal-related regulated cell death modalities—in a spatial context. It does not imply that ferroptosis and cuproptosis constitute one experimentally unified pathway or a single recognized RCD category. This axis uniquely integrates metabolic zonation, oxygen gradients, and metal-dependent cell death into a cohesive spatial framework.

In recent years, breakthroughs in spatially resolved omics technologies have provided unprecedented tools for deciphering hepatic zonal heterogeneity. Single-cell RNA sequencing (scRNA-seq) combined with spatial transcriptomics has successfully generated high-resolution maps of gene expression across the liver lobule, revealing the continuous distribution of metabolic enzymes, transporters, and antioxidant molecules from Zone 1 to Zone 3 [16,17]. In addition, novel imaging techniques, such as multibeam imaging, enable the integrated analysis of elements such as iron in liver tissue sections at nanoscale spatial, chemical, and isotopic dimensions, allowing direct visualization of complex zonal regulatory mechanisms following iron overload [18]. Concurrently, the application of fluorescent probes permits dynamic imaging of ferroptosis levels in situ within tissues [19]. Collectively, these technological advances are driving a long-overdue paradigm shift in liver research: from viewing the liver as a homogeneous entity to the functionally compartmentalized zoned ecosystem.

Ferroptosis and cuproptosis, two emerging forms of metal-dependent RCD, are rapidly becoming focal points in the studies of various liver diseases. Ferroptosis, characterized by glutathione (GSH) depletion, glutathione peroxidase 4 (GPX4) inactivation, and lipid peroxidation, is widely implicated in the pathological processes of alcohol-associated liver disease [20], drug-induced liver injury (DILI), liver fibrosis, and HCC [21]. Cuproptosis, which depends on copper ion-induced aggregation of mitochondrial lipoylated proteins and damage to iron-sulfur cluster proteins, is implicated in the pathogenesis of MASLD [22]. Notably, these two RCD modalities involve multiple core molecular hubs, including the GSH antioxidant system, mitochondrial function, and nuclear factor erythroid 2-related factor 2 (NRF2)/tumor protein p53 signaling pathways [23,24,25]. More importantly, iron and copper homeostasis are not isolated. Ceruloplasmin (CP), a ferroxidase, intimately links copper metabolism to iron export. This raises the hypothesis that the two metals may be spatially coupled and form a synergistic “spatial redox–metalloptosis axis” [26].

Based on these observations, this review proposes and elaborates on the “spatial redox–metalloptosis axis” hypothesis: distinct zones of the liver, owing to inherent gradients in metabolism, redox status, and metal homeostasis, result in differential susceptibility to ferroptosis and cuproptosis. Specifically, pericentral Zone 3 is hypothesized to be predisposed to ferroptosis because of hypoxia, a pro-ferroptotic microenvironment (rather than absolute iron enrichment), and relatively weak antioxidant capacity, whereas periportal Zone 1 is hypothesized to be more susceptible to cuproptosis because of the enrichment of copper transporters and specific metabolic characteristics.

## 2. The Blueprint of the Liver: Spatial Zonation of Metabolism, Redox, and Metals

### 2.1. The Weft and Warp of Metabolism: From Classical Zonation to Single-Cell Atlases

**Figure 1:**
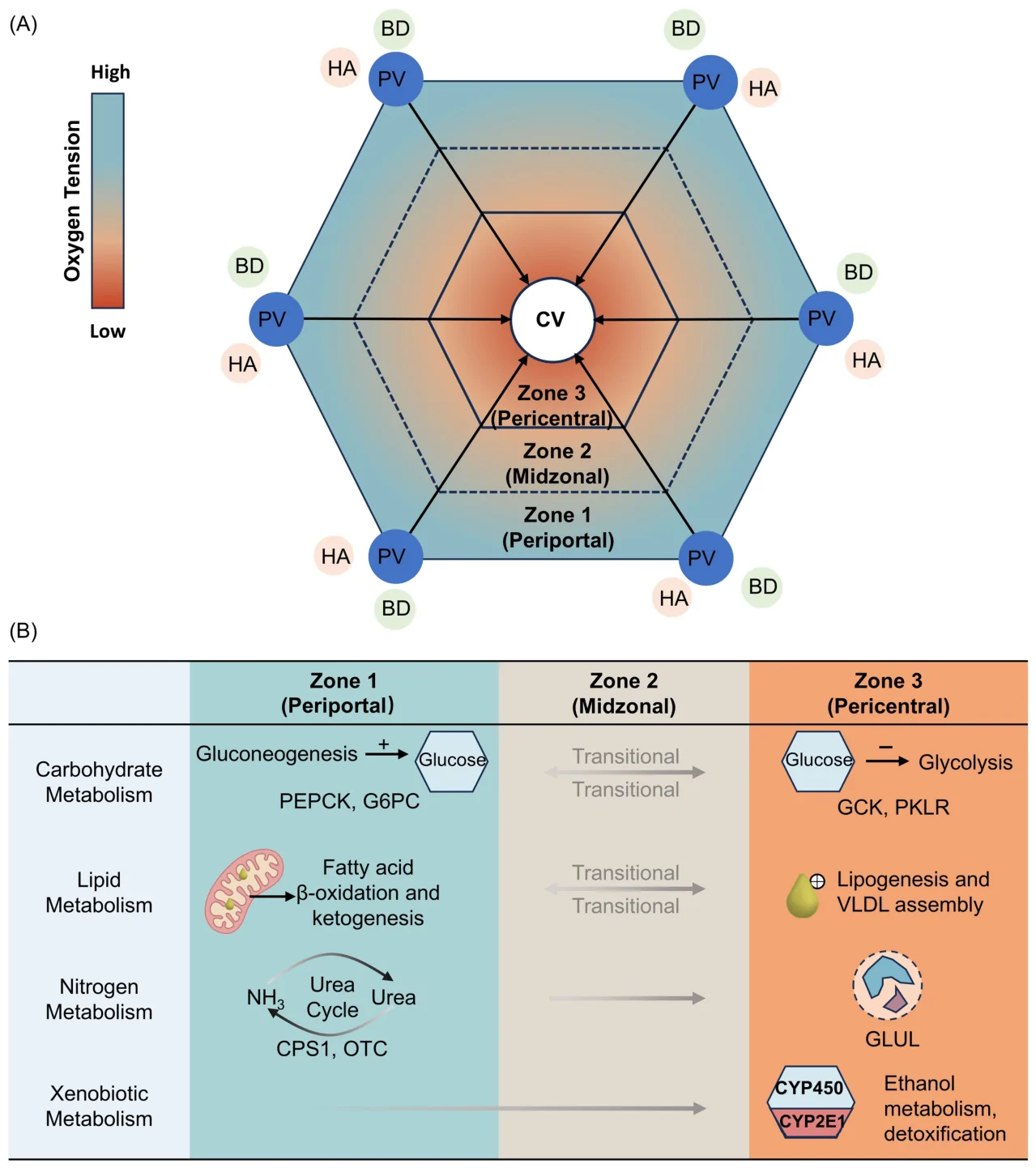
Liver metabolic zonation. (**A**) Classical hexagonal lobule with 3 functional zones along the porto-central axis, forming oxygen/nutrient gradients. The background color gradient represents the oxygen tension gradient in the hepatic parenchyma, which decreases from 60–65 mmHg (high oxygen, periportal Zone 1) to 30–35 mmHg (low oxygen, pericentral Zone 3) [34]. These values may vary across species, experimental conditions, and disease states and should not be interpreted as universal fixed values. All vessels and ducts are labeled with standard anatomical colors independent of the oxygen gradient: portal vein (PV, blue dot), hepatic artery (HA, orange dot), bile duct (BD, green dot), central vein (CV, black outline with white fill). (**B**) Zonal distribution of core metabolic pathways and key molecular markers. Abbreviations: PV, portal vein; HA, hepatic artery; BD, bile duct; CV, central vein; PEPCK, phosphoenolpyruvate carboxykinase; G6PC, glucose-6-phosphatase catalytic; GCK, glucokinase; PKLR, pyruvate kinase L/R; VLDL, very low-density lipoprotein; CPS1, carbamoyl phosphate synthetase 1; OTC, ornithine transcarbamylase; GLUL, glutamate-ammonia ligase; CYP450, cytochrome P450; CYP2E1, cytochrome P450 family 2 subfamily E member 1.

The remarkable functional complexity of the liver is inseparable from its intricate structural organization. The classical hepatic lobule model is divided into three functional zones: the periportal zone (Zone 1), the midzonal zone (Zone 2), and the pericentral, or perivenous, zone (Zone 3). Physiologically, nutrient-rich blood enters from Zone 1, sequentially flows through the zones, and exits via Zone 3, establishing a radial metabolic gradient [27]. The concentrations of oxygen, nutrients, and hormones exhibit significant gradients along this axis, driving the compartmentalization of metabolic functions [28]. Specifically, Zone 1, which is rich in oxygen and nutrients, predominantly governs energy-demanding processes such as gluconeogenesis, fatty acid β-oxidation, and the urea cycle. In contrast, Zone 3, with its relatively hypoxic environment, primarily undergoes reductive reactions such as glycolysis, lipogenesis, and drug metabolism [29]. The zonal distribution of carbohydrate metabolism is particularly archetypal. Gluconeogenic enzymes (phosphoenolpyruvate carboxykinase; glucose-6-phosphatase catalytic) are located mainly in Zone 1. In contrast, glycolytic enzymes (glucokinase; pyruvate kinase L/R) are enriched in Zone 3 [30]. Lipid metabolism also displays similar zonal segregation: fatty acid oxidation and ketogenesis tend to occur in Zone 1, whereas lipogenesis and very-low-density lipoprotein assembly are concentrated in Zone 3 [31]. Nitrogen metabolism is even more distinctly zoned: urea cycle enzymes (carbamoyl phosphate synthetase 1; ornithine transcarbamylase) are confined to Zone 1, whereas glutamine synthetase (glutamate-ammonia ligase, GLUL) is expressed only in a narrow ring of hepatocytes surrounding the central vein, serving as a classic marker of Zone 3 identity [32]. Xenobiotic metabolism—the liver’s detoxification machinery—is also highly zonated: the cytochrome P450 (CYP450) enzyme family, particularly the major ethanol-metabolizing enzyme cytochrome P450 family 2 subfamily E member 1 (CYP2E1), is selectively expressed in Zone 3 [33] (Figure 1).

In recent years, the advent of single-cell and spatial omics technologies has profoundly improved our understanding of this metabolic zonation. More than 60 zonated genes have been identified by reconstructing the spatial atlas of the mouse liver [16]. It should be noted that the three-zone model is a useful simplification; modern liver zonation is increasingly recognized as a continuous gradient rather than strictly discrete compartments. Zone 2 is not merely a transitional area but a distinct compartment with a unique transcriptional identity [35,36]. Complementing these spatial atlas findings, scRNA-seq studies have identified 94 hepatocyte-specific zonation markers, of which 39 are unique to Zone 3 and 55 are unique to Zone 1 [37]. Spatial transcriptomics in healthy human liver has further revealed four transcriptionally distinguishable hepatocyte subpopulations, providing a high-resolution map of hepatic lobule spatial heterogeneity [38]. Furthermore, matrix-assisted laser desorption/ionization imaging mass spectrometry and isotope tracing directly visualized spatial metabolite gradients in the mouse liver. Specifically, tricarboxylic acid (TCA) cycle intermediates, glutamine, and lactate-derived labels are concentrated mainly in Zone 1, whereas ATP depletion due to fructose metabolism occurs specifically in Zone 3 [39]. These findings not only validate the classical zonation model but also reveal the precise arrangement of metabolic pathways at the microscopic level. Notably, this zonation exhibits dynamic adaptability—during fasting, gluconeogenic gene expression progressively expands from Zone 1 to Zone 3, indicating that metabolic function can undergo spatiotemporal reprogramming in response to nutritional status [40]. Organoid models have also successfully recapitulated the regional differentiation trajectory of the human liver; by modulating factors such as E1A binding protein p300/ten-eleven translocation 1 or hypoxia-inducible factor 1α, hepatocytes with periportal, midzonal, and pericentral identities can be constructed in vitro [41]. It is crucial to note that the zonal pattern of steatosis in MASLD is age-dependent. While adult MASLD typically initiates with Zone 3 macrovesicular steatosis [42], pediatric MASLD frequently presents with a more diffuse or even Zone 1-predominant fat distribution pattern [43]. This spatial discrepancy underscores the complexity of metabolic programming and suggests that spatial metalloptosis profiles might also differ across age groups. In summary, from classical anatomy to single-cell atlases, the spatial logic of hepatic metabolism has advanced from macroscopic description to molecular mechanistic resolution.

### 2.2. Redox Gradients: The Oxygen Paradox and Mismatched Antioxidant Defenses

The periportal–pericentral oxygen gradient profoundly influences hepatic redox homeostasis. Blood flow from Zone 1 toward Zone 3 results in progressive oxygen consumption by hepatocytes, establishing a decreasing oxygen partial pressure gradient, with higher oxygen partial pressure in Zone 1 (approximately 60–65 mmHg) and a relatively hypoxic environment in Zone 3 (30–35 mmHg) [34]. A thought-provoking paradox—that regions with high metabolic activity may face higher oxidative stress—forms the basis of hepatic redox zonation. Although Zone 3 is relatively hypoxic, its enrichment in CYP450 enzymes (particularly CYP2E1) and high respiratory activity in pericentral mitochondria lead to increased basal reactive oxygen species (ROS) generation [44]. Meanwhile, when the oxygen supply is ample in Zone 1, it performs high-energy-demanding metabolic tasks (e.g., β-oxidation and gluconeogenesis) [39]; indeed, the oxygen consumption rate of Zone 1 hepatocytes is approximately four times that of Zone 3 hepatocytes [45], thus also facing oxidative pressure. However, the distribution of the antioxidant defense system does not perfectly match the levels of oxidative stress and is spatially organized. For instance, GSH, the liver’s primary redox buffer, has its key synthesis enzymes, glutamate–cysteine ligase catalytic subunit and glutamate–cysteine ligase modifier subunit, that are more highly expressed in Zone 1 [46]. Thus, Zone 3 displays a reduced capacity for glutathione synthesis. This statement refers specifically to the synthetic capacity rather than the total regional GSH abundance, and the direct relationship between this synthetic gradient and ferroptosis susceptibility in human liver remains to be fully validated. Conversely, Zone 3 hepatocytes exhibit high expression of GLUL to maintain intracellular glutamine pools. Glutamine serves as an important precursor for glutamate production, which is in turn a substrate for GSH synthesis. This represents an indirect contribution to antioxidant capacity, rather than direct antioxidant enzyme activity [47]. This regional disparity in defensive capacity may render specific zones more prone to imbalance under stress conditions. Microfluidic chip models simulating the hepatic oxygen gradient revealed that as the oxygen concentration decreases, hypoxia-inducible factor 1α nuclear translocation increases in HepG2 cells, accompanied by a significant decrease in albumin expression [48]. This suggests that hypoxia affects metabolic function and may impair the synthesis capacity of antioxidant proteins. In MASLD, fat deposition and fibrosis further disrupt the native oxygen gradient, exacerbating localized hypoxia and forming a vicious cycle; chronic intermittent hypoxia (e.g., sleep apnea) has also been confirmed to be closely related to MASLD progression, highlighting the importance of intrahepatic hypoxia as a spatial determinant [49]. Furthermore, tumors can systemically disrupt hepatic zonation, selectively impairing the zonal pattern of detoxification metabolic genes while preserving some energy metabolism pathways, suggesting that the regional vulnerability of redox homeostasis may be exploited by pathological processes [50]. Therefore, the redox gradient is not merely a consequence of physiological zonation but also a critical variable determining regional susceptibility to injury.

### 2.3. Metal Territories: Spatial Distribution of Iron and Copper Transporters

In the context of this hypothesis-driven review, we primarily discuss the functional flux and transporter expression of metals, rather than absolute total tissue concentrations, to reflect their biological availability and relevance to cell death vulnerability. As essential transition metals for life, iron and copper also follow strict spatial rules in their hepatic distribution, with the zonal expression of their transporters shaping the regional preference for metalloptosis, including ferroptosis and cuproptosis. Researchers using X-ray fluorescence microscopy have observed spatial gradients of iron and copper within the hepatic lobule, which are altered upon toxicant exposure [51]. We acknowledge that in the healthy liver, total iron concentration lacks a steep absolute gradient across the lobule. Furthermore, in pathological states like MASLD, absolute iron deposition can be mixed or even periportal (Zone 1). However, our hypothesis posits that it is the microenvironmental vulnerability of Zone 3, rather than absolute iron enrichment, that drives its susceptibility to ferroptosis. The pericentral zone (Zone 3) is characterized by relative hypoxia, robust CYP2E1 activity [27,33], higher lipid accumulation (in adult MASLD) [42], and relatively weaker GSH synthesis capacity [46]. This unique metabolic combination creates a highly sensitized “pro-ferroptotic niche”. The molecular mechanism of ferroptosis, where iron catalyzes lipid peroxidation of polyunsaturated fatty acids, is well-established [52]. Thus, when iron is present (even at normal or moderately elevated levels), the unique microenvironment of Zone 3 creates a permissive setting for ferroptosis initiation. However, the classical execution mechanism of ferroptosis relies on lipid peroxidation, and Zone 3, with its high expression of pro-oxidant enzymes such as CYP450 [53] and relatively weak antioxidant capacity, may be more prone to triggering the ferroptosis cascade.

Regarding copper, direct evidence for absolute copper concentration gradients in the healthy liver is limited. However, the functional spatial bias is well-established and driven by the portal “first-pass effect”. Portal blood delivers copper initially to Zone 1 hepatocytes, which are responsible for the majority of hepatic copper uptake; however, first-pass exposure does not necessarily equate to higher steady-state intracellular copper levels in Zone 1, as copper may be subsequently redistributed, stored, or exported. This continuous functional flux makes Zone 1 intrinsically more exposed to copper toxicity dynamics [27]. Copper is finely regulated by transporters such as ATPase copper transporting beta (ATP7B) [54]. Furthermore, the cellular landscape of metal homeostasis involves non-parenchymal cells. Kupffer cells, the resident macrophages of the liver, tend to be enriched in the periportal area (Zone 1) [55]. However, this macrophage distribution should not be conflated with hepatocyte ferroptosis susceptibility: Kupffer-cell iron recycling occurs in macrophages, while ferroptosis susceptibility in this review refers to hepatocytes. The relationship between periportal macrophage iron handling and periportal hepatocyte ferroptosis remains to be directly established. They play a central role in iron recycling by phagocytosing senescent red blood cells and regulating local iron availability [56]. Under inflammatory conditions, activated Kupffer cells can release significant amounts of iron and cytokines, which disrupt local metal homeostasis and interact with the zonal susceptibility of adjacent hepatocytes, further shaping the spatial pattern of metalloptosis. The precise intralobular localization of ATP7B, the copper efflux protein that is mutated in Wilson’s disease, has yet to be fully elucidated, but existing evidence suggests that ATP7B is translocated to the canalicular membrane in response to copper concentration, where it participates in biliary copper excretion [57,58].

**Figure 2:**
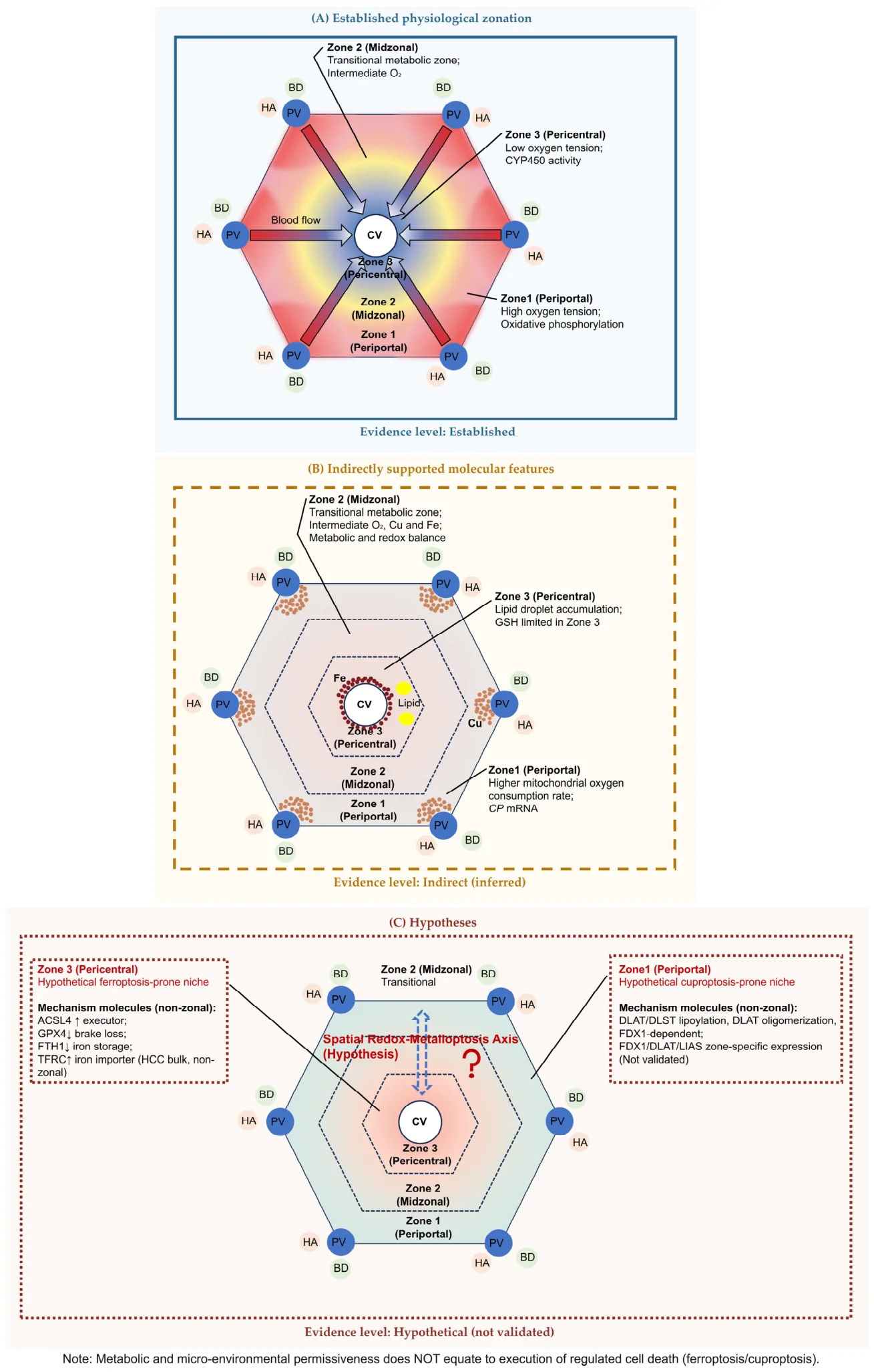
Proposed integrated model of copper–iron zonation, metabolic features, and metalloptosis susceptibility along the hepatic lobule. The figure categorizes evidence into three distinct panels: (**A**) established physiological zonation (solid border); (**B**) indirectly supported molecular features (dashed border). “Fe” and “Cu” designate inferred metal handling and distribution features, not specific ionic states; (**C**) untested hypotheses (dotted border). The hexagon represents a hepatic lobule cross-section; the radial gradient indicates oxygen tension and blood flow direction. The arrow with a question mark denotes the unvalidated “spatial redox–metalloptosis axis”. Abbreviations: ACSL4, acyl-CoA synthetase long-chain family member 4; BD, bile duct; CP, ceruloplasmin; CV, central vein; CYP450, cytochrome P450; DLAT, dihydrolipoamide S-acetyltransferase; DLST, dihydrolipoamide S-succinyltransferase; FDX1, ferredoxin 1; FTH1, ferritin heavy chain 1; GPX4, glutathione peroxidase 4; GSH, glutathione; HA, hepatic artery; LIAS, lipoic acid synthetase; PV, portal vein; TFRC, transferrin receptor. Note: Metabolic permissiveness does NOT equate to execution of regulated cell death (ferroptosis/cuproptosis).

Additionally, species differences are a crucial consideration when interpreting these zonal maps. While the overall pattern of metabolic zonation is broadly conserved between mice and humans, notable species-specific differences exist. For example, the pericentral enrichment of certain metabolic enzymes, such as CYP2E1, often exhibits different gradients and expression scales in human livers compared to murine ones [33]. Conversely, the “first-pass effect” of portal copper delivery remains anatomically conserved [27]. We emphasize that these inter-species variations must be carefully considered when translating findings regarding spatial metal metabolism from murine models to human pathology. CP deserves special attention as a molecular bridge connecting copper and iron metabolism [59]. CP is a copper-containing ferroxidase that oxidizes Fe^2+^ to Fe^3+^, a necessary step for iron export via ferroportin and subsequent binding to transferrin [60]. Its synthesis requires copper incorporation, indicating that CP activity serves as both a direct readout of copper status and a key determinant of iron efflux. In metabolic dysfunction-associated steatohepatitis (MASH) patients and animals, the expression of hepatocyte-derived CP is significantly upregulated. Specifically, the knockout of hepatocyte CP alleviates steatosis, inflammation, and fibrosis and remodels bile acid metabolism by upregulating the expression of cytochrome P450 family 7 subfamily A member 1 and cytochrome P450 family 8 subfamily B member 1 [61] (Figure 2).

## 3. Ferroptosis: A Susceptible Phenotype of the Pericentral Zone

### 3.1. Molecular Execution and Regulation of Ferroptosis

Throughout this review, we acknowledge that the reported ferroptosis phenotypes in liver disease are largely based on inferred pathway activation (e.g., lipid peroxidation, GSH depletion, GPX4 reduction) rather than spatially resolved genetic or pharmacological rescue experiments. Unless explicitly noted, these observations should be interpreted as ferroptosis susceptibility rather than definitively executed cell death. Ferroptosis is an iron-dependent, non-apoptotic form of RCD characterized centrally by the aberrant accumulation of lipid peroxides and dysfunction of the antioxidant defense system [62]. This process is driven by ferrous iron catalysis of the peroxidation of polyunsaturated fatty acids via the Fenton reaction, ultimately leading to loss of cell membrane integrity and cell lysis [63]. At the molecular level, the system Xc^−^-GSH-GPX4 axis constitutes the core inhibitory pathway of ferroptosis. System Xc^−^, composed of solute carrier family 7 member 11 (SLC7A11/xCT) and solute carrier family 3 member 2, mediates cystine uptake for GSH synthesis, whereas GPX4 utilizes GSH to reduce lipid hydroperoxides to nontoxic alcohols, thus blocking the lipid peroxidation chain reaction [64]. When this pathway is compromised (e.g., downregulation of SLC7A11 or GPX4), cells fail to effectively clear lipid ROS and trigger ferroptosis [65]. Other defense mechanisms include the ferroptosis suppressor protein 1-coenzyme Q10 system, the dihydroorotate dehydrogenase-mediated mitochondrial antioxidant pathway, and the guanosine triphosphate cyclohydrolase 1-tetrahydrobiopterin axis [66]. Dysregulated iron metabolism is a key driver of ferroptosis. Expansion of the labile iron pool can release stored iron via nuclear receptor coactivator 4 (NCOA4)-mediated ferritinophagy, further exacerbating lipid peroxidation [67]. Epigenetic and posttranslational modifications dynamically regulate the expression and stability of ferroptosis-related genes. For instance, tripartite motif containing 34 stabilizes *GPX4* mRNA by promoting regulator of nonsense transcripts 1 degradation and enhancing ferroptosis resistance in cells [68]. Conversely, methyltransferase-like 9 promotes ferroptosis by downregulating *SLC7A11* mRNA expression [69]. NRF2, a core transcription factor, upregulates the expression of multiple antioxidant genes, including heme oxygenase 1, ferritin heavy chain 1 (*FTH1*), and *SLC7A11*, constituting the primary line of defense against oxidative stress [70].

### 3.2. Evidence of Ferroptosis in Liver Diseases: A Diagnosis of Zonal Blindness

Although ferroptosis has been widely implicated in the pathological progression of various liver diseases, including MASH, liver fibrosis, cirrhosis, HCC, DILI, and ischemia-reperfusion injury, most existing studies overlook its spatial distribution, resulting in significant “zonal blindness”. In liver samples from patients and animals, ferroptosis-associated molecular changes are frequently observed, and iron chelators or ferroptosis inhibitors (e.g., ferrostatin-1 and liproxstatin-1) can effectively ameliorate liver injury [71]. It should be emphasized that none of these alterations—including reduced GPX4 expression, lipid-peroxidation products, or iron accumulation—is individually diagnostic of ferroptotic cell death, and robust claims require rescue-based validation. In liver fibrosis, ferroptosis plays a dual role: it can inhibit fibrosis by inducing death in activated HSCs, but it can also cause hepatocyte death and indirectly activate profibrotic signals [11]. In human cirrhotic tissue, GPX4 expression is significantly downregulated in fibrotic areas, and chronic iron dextran administration in mouse models induces ferroptosis accompanied by increased alpha-smooth muscle actin and plasminogen activator inhibitor-1 expression [21]. In HCC, tumor cells often acquire resistance to ferroptosis by upregulating GPX4 or SLC7A11 expression, thereby promoting proliferation and metastasis [72]. Notably, recent spatially resolved techniques are beginning to reveal the zonal specificity of ferroptosis. A spatially resolved study published in Science demonstrated that Zone 3 hepatocytes, which are endowed with unique antioxidant capacity and ferroptosis resistance, are more likely to serve as cells of origin for tumors. High expression of glutathione S-transferase mu 2/3 in Zone 3 helps cells resist ferroptosis by reducing lipid peroxidation and increasing GSH levels, thus increasing survival in the context of oncogenic mutations [73]. However, the vast majority of mechanistic studies still rely on whole-liver homogenates or nonzonated cell models and fail to resolve the precise spatial map of ferroptosis within the hepatic lobule.

### 3.3. Mechanistic Hypothesis for Pericentral Susceptibility to Ferroptosis

The liver exhibits pronounced metabolic zonation, with Zone 3 hepatocytes chronically exposed to a hypoxic environment and tasked with high burdens of drug metabolism and lipid processing, placing them in a state of elevated redox pressure [27]. This zone features active iron metabolism and relatively weak antioxidant defenses, potentially forming the basis for hypothesized susceptibility to ferroptosis. In MASLD animals, ferroptosis is accompanied by decreased expression of FTH1 and GPX4, and increased expression of Acyl-CoA synthetase long-chain family member 4 (ACSL4) [74]. NCOA4-mediated ferritinophagy can increase the labile iron pool and promote lipid peroxidation, and a study revealed that V3, a proteolysis-targeting chimera-based NCOA4 degrader, effectively inhibits ferroptosis and alleviates liver injury [67]; these results suggest that NCOA4 may play a critical role in pericentral ferroptosis susceptibility. Furthermore, the transcription factor EB-NCOA4 axis has been shown to mediate hepatocyte ferroptosis induced by polyether ionophore antibiotics, further supporting the driving role of ferritinophagy in pericentral ferroptosis [75]. The hypoxic environment in Zone 3 may impair the activation efficiency of antioxidant pathways such as NRF2; however, previous research has suggested that fibroblast growth factor 21 can inhibit ferroptosis by promoting ubiquitination of heme oxygenase 1 and activating NRF2, while its deficiency exacerbates iron overload-induced liver injury [76]. While recent spatially resolved proteomics studies have provided powerful frameworks for mapping human liver zonation [47], direct evidence for the specific intralobular distribution of ferroptosis-related proteins such as ACSL4, NCOA4, NRF2, FTH1, and GPX4 is currently lacking. Consequently, based on bulk expression data and known metabolic zonation patterns, it is inferred that the pericentral zone has higher ACSL4 and NCOA4 protein expression, and lower NRF2, FTH1, and GPX4 expression; however, this inference requires direct zone-resolved validation. We acknowledge that oncogenic mutations (e.g., initiating HCC) represent a critical context in which Zone 3 hepatocytes can mount robust antioxidant programs to resist ferroptosis [73]. This finding is integrated into our central model as the primary example of context-dependent modulation: the hypothesized Zone 3 baseline susceptibility applies to non-oncogenic acute/chronic metabolic liver injury (the primary focus of this review), while oncogenic transformation represents a distinct pathological context that overrides baseline zonal vulnerability.

### 3.4. Summary and Research Gaps for Ferroptosis

Ferroptosis has been established as a key pathogenic mechanism in various liver diseases, with its molecular execution pathway involving complex interactions among iron metabolism, lipid peroxidation, and antioxidant defense systems. Accumulating evidence supports the hypothesis that Zone 3 is a candidate zone of increased susceptibility under selected stress conditions for ferroptosis, closely related to its unique metabolic and redox microenvironment. However, the vast majority of current studies still employ bulk tissue analysis, lacking fine-resolution dissection of intralobular spatial heterogeneity. This leaves the “pericentral susceptibility” hypothesis largely unvalidated by systematic study. Furthermore, whether ferroptosis exhibits zonal preference uniformly across different liver diseases (e.g., viral hepatitis, DILI, and hereditary iron overload disorders) and the differential responses to ferroptosis among various cell types (e.g., hepatocytes, Kupffer cells, and HSCs) in the spatial dimension remain to be thoroughly investigated. Resolving the apparent contradiction between basal ferroptosis resistance in oncogenic contexts and pathological vulnerability in injury contexts is a key priority for future research. To address these gaps, several well-established zone-specific genetic manipulation tools could be employed. For pericentral (Zone 3) targeting, transgenic models employing the *Cyp2e1* promoter to drive Cre recombinase expression provide reliable tools for manipulating gene expression in hepatocytes surrounding the central vein [77]. For viral vector-based approaches, although tail-vein injected adeno-associated virus serotype 8 vectors exhibit pan-hepatocyte tropism, spatial specificity can be enhanced by incorporating zone-specific promoters [27]. For example, vectors driven by the *Cyp2e1* promoter can preferentially target Zone 3 hepatocytes, while those driven by the carbamoyl phosphate synthetase 1 promoter can be used for Zone 1 targeting. These approaches provide concrete strategies to experimentally test the zonal susceptibility hypotheses in vivo. Future research urgently needs to integrate spatial transcriptomics, imaging mass cytometry, and zone-specific genetic manipulation techniques to map the precise ferroptosis landscape within the hepatic lobule, filling the current knowledge gap of “zonal blindness”.

## 4. Hypothesized Periportal Susceptibility to Cuproptosis

### 4.1. Molecular Mechanism of Cuproptosis

We distinguish two categories of cuproptosis evidence in this review: (1) cuproptosis-related gene signatures (transcriptomic/bioinformatic associations, which do not establish that cuproptosis occurs biologically); (2) mechanistically demonstrated cuproptosis (requires lipoylated protein aggregation, Fe-S cluster protein destabilization, and rescue by copper chelation or genetic disruption). It is important to note that the vast majority of studies cited regarding cuproptosis in MASLD, ALD, or HCC derive from tissue copper measurements, pathway-associated transcriptomics, or cell or animal models. Currently, direct spatial evidence and zone-specific rescue validation are almost entirely absent. Cuproptosis is a distinct form of RCD triggered by intracellular copper overload, whose core mechanism relies on the direct binding of copper ions to lipoylated enzymes of the TCA cycle [78]. This binding induces disulfide bond-dependent abnormal aggregation of lipoylated proteins, leading to proteotoxic stress and destabilization of iron—sulfur cluster proteins, ultimately disrupting mitochondrial function and triggering cell death [79]. This process predominantly occurs in cells that are highly dependent on oxidative phosphorylation for energy, as they are more sensitive to the integrity of the TCA cycle [80]. Key regulators include ferredoxin 1 (FDX1), a crucial reductase for lipoylation modification, which not only participates in the transfer of lipoyl groups but also mediates the binding of copper ions to lipoylated proteins. In addition, TCA cycle enzymes such as dihydrolipoamide S-acetyltransferase (DLAT) are primary targets for copper binding and aggregation [81]. p53 potentially indirectly influences cuproptosis sensitivity by promoting oxidative phosphorylation, inhibiting glycolysis, and regulating the biosynthesis of GSH and iron-sulfur clusters [80]. Notably, cuproptosis is not driven primarily by ROS but is characterized by copper binding-induced aberrant aggregation of lipoylated proteins and mitochondrial proteotoxic stress, distinguishing it from other death mechanisms, such as ferroptosis, apoptosis, and necrosis [82].

### 4.2. Preliminary Evidence of Hepatic Cuproptosis

Several recent studies have provided experimental evidence for the occurrence of cuproptosis in liver diseases. It is important to distinguish between mechanistic demonstrations and bioinformatic associations. We classify cuproptosis-related evidence into three levels: (1) direct mechanistic validation via genetic or pharmacological perturbation of the cuproptosis machinery; (2) indirect detection of core molecular markers (such as lipoylated protein aggregation and Fe-S cluster loss); and (3) associative bioinformatic analyses based on gene expression, prognostic signatures, or computational models. Associative evidence does not constitute direct proof of cuproptosis pathway activation.

In HCC, copper ionophores (Evidence type: cell and animal model) have been shown to increase copper uptake, inducing DLAT aggregation, depleting GSH, and triggering oxidative stress, thereby demonstrating direct functional activation of cuproptosis and antitumor effects in HCC cell lines and animal models [83]. In contrast, bioinformatic analyses based on The Cancer Genome Atlas and Gene Expression Omnibus databases have identified that multiple cuproptosis-related genes (including glutaminase, cyclin-dependent kinase inhibitor 2A, *DLAT*, *FDX1*, and lipoic acid synthetase) are aberrantly expressed in HCC and are associated with poor prognosis and an immunosuppressive microenvironment [84]. These findings represent associative evidence and do not confirm the activation of the cuproptosis pathway. In MASLD or MASH, the cuproptosis pathway is also activated (Evidence type: pathway-associated transcriptomics): the expression of cuproptosis-related genes such as dihydrolipoamide dehydrogenase and pyruvate dehydrogenase E1 subunit beta is significantly upregulated in patient liver tissues and high-fat diet mouse models, and their expression is positively correlated with hepatic steatosis and disease activity scores [85]. However, these associations are bioinformatic or correlative, and do not directly demonstrate that cuproptosis is actively occurring. In a hepatic ischemia-reperfusion injury model, a diagnostic model constructed from eight differentially expressed cuproptosis-related genes achieved a high area under the curve of 0.97 [86], indicating a strong clinical association rather than directly proving that cuproptosis mediates the injury. Of particular interest, allicin derivatives have been mechanistically validated to selectively trigger cuproptosis in HSCs by inducing ras-related protein Rab-18 (RAB18) phase separation and promoting succinylation at lysine 320 of dihydrolipoamide dehydrogenase, thereby exerting therapeutic effects in fibrosis [87]. Furthermore, in cholestatic liver injury, taurocholic acid promotes hepatic copper accumulation, leading to the downregulation of FDX1 expression, which in turn impairs DLAT lipoylation function and induces cuproptosis predominantly in Zone 1 [88]. This represents a mechanism-based demonstration of zone-specific cuproptosis.

### 4.3. Mechanistic Hypothesis for Periportal (Zone 1) Susceptibility to Cuproptosis

Although direct evidence definitively proving an absolute predilection of cuproptosis for Zone 1 is currently lacking, several indirect findings support its zonal preference. First, hepatic hemodynamics dictate that portal blood, which is rich in nutrients and potentially toxic substances, first enters Zone 1, exposing hepatocytes in this region to high concentrations of exogenous copper ions chronically [27,89]. Second, scRNA-seq and spatial metabolomics data demonstrate that periportal hepatocytes possess unique metabolic characteristics, including highly active oxidative phosphorylation and a robust TCA cycle [77,90]. Since cuproptosis fundamentally depends on copper-induced aggregation of lipoylated mitochondrial proteins within the TCA cycle [79], Zone 1 is hypothesized to have a lower threshold for cuproptosis initiation due to its high oxidative phosphorylation activity and TCA-cycle flux. Additionally, periportal hepatocytes exhibit specific transcriptional activation (e.g., periportal inflammatory hepatocytes subpopulation) under inflammatory conditions. For example, during endotoxemia, they facilitate macrophage recruitment via the C-C motif chemokine ligand 2-C-C motif chemokine receptor 2 pathway and upregulate programmed death-ligand 1 expression to form an immunosuppressive microenvironment [91]. Meanwhile, cuproptosis has been shown to be correlated with immune infiltration status [92]. In summary, we hypothesize that Zone 1 may represent a permissive microenvironment for cuproptosis (proposed hypothesis; direct zone-resolved evidence of cuproptosis execution in Zone 1 is currently absent). This is particularly relevant in chronic liver diseases such as MASLD, MASH, liver fibrosis, and ischemia-reperfusion injury. The vulnerability of Zone 1 is attributed to its unique metabolic phenotype, blood flow exposure characteristics, and immune microenvironment.

### 4.4. Summary and Research Gaps of Cuproptosis

In conclusion, Zone 1 is hypothesized to possess multiple features that may increase permissiveness to hypothesized cuproptosis: high copper flux, coexposure to bile acids, and potentially elevated copper uptake (ceruloplasmin synthesis is proposed as a candidate link but does not itself demonstrate increased hepatocellular copper uptake). However, direct evidence is almost entirely absent. Current critical research gaps include a lack of spatially resolved evidence confirming the precise intralobular distribution of cuproptosis markers. Second, the expression atlas of copper transporters across different liver zones has not been systematically mapped. Third, no zone-targeted intervention strategies have been developed. Crucially, direct spatial evidence connecting these biochemical features to the actual execution of cuproptosis in Zone 1 is currently almost entirely absent. To facilitate a systematic assessment of evidence strength, we have summarized the key zonal claims for ferroptosis and cuproptosis and their supporting evidence in Table 1 and Table S1.

## 5. The Spatial Redox–Metalloptosis Axis: Molecular and Spatial Interactions

### 5.1. Shared Molecular Hubs

**Figure 3:**
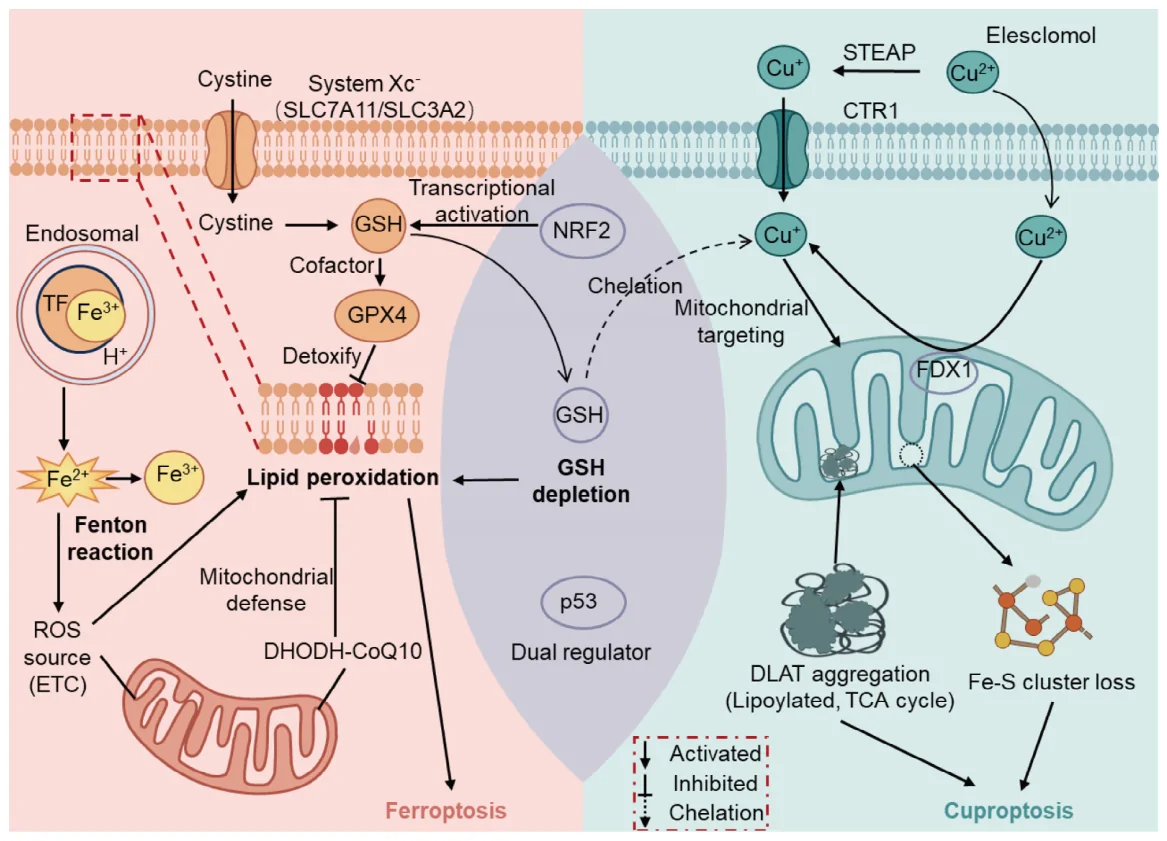
Shared molecular hubs between ferroptosis and cuproptosis. Depicts core molecular pathways of ferroptosis and cuproptosis, highlighting their shared regulatory hubs: GSH depletion, mitochondrial dysfunction, and NRF2/p53-mediated transcriptional regulation. Abbreviations: GSH, glutathione; GPX4, glutathione peroxidase 4; TF, transferrin; ROS, reactive oxygen species; ETC, electron transport chain; DHODH, dihydroorotate dehydrogenase; CoQ10, coenzyme Q10; STEAP, six-transmembrane epithelial antigen of the prostate; FDX1, ferredoxin 1; DLAT, dihydrolipoamide S-acetyltransferase; TCA, tricarboxylic acid; NRF2, nuclear factor erythroid 2-related factor 2.

Although ferroptosis and cuproptosis are distinct forms of RCD, they exhibit significant mechanistic interactions at the molecular level. Both of them are highly dependent on the dysregulation of redox homeostasis, mitochondrial dysfunction, and regulation by key signaling proteins [97]. Mitochondrial integrity is a critical structural foundation shared by both. In ferroptosis, mitochondria serve as both a source of ROS (via the electron transport chain) and an important site of defense (via dihydroorotate dehydrogenase-mediated coenzyme Q10 reduction) [98]. In cuproptosis, mitochondria are the primary target, with lipoylated TCA cycle enzymes localized within them, and copper-induced protein aggregation directly impairs their function [79]. Therefore, mitochondrial dysfunction is a common feature of these two death modalities. In addition, GSH acts as a shared metabolic node and plays a central role in both pathways. In ferroptosis, GSH is an essential cofactor for GPX4; moreover, depletion of GSH leads to GPX4 inactivation and uncontrolled lipid peroxidation [99]. In cuproptosis, GSH serves as the primary intracellular copper chelator; its depletion can lead to the accumulation of free copper ions, thereby triggering the death program [79]. The lower GSH levels in Zone 3 [100] may render it more sensitive to both forms of cell death [90]. Furthermore, transcription factors such as NRF2 (a common inhibitor of ferroptosis and cuproptosis) and p53 (a dual regulator of both) have been confirmed to simultaneously regulate the expression of genes related to ferroptosis and cuproptosis and play important roles in HCC therapy [23] (Figure 3). We clarify that shared regulatory pathways (e.g., GSH, NRF2, p53, mitochondrial dysfunction) indicate conceptual convergence but do not necessarily establish direct biological crosstalk between ferroptosis and cuproptosis; direct mechanistic interaction remains to be demonstrated.

### 5.2. A Proposed Molecular Bridge Connecting Copper and Iron: CP as a Forward-Looking Hypothesis

CP, as a copper-containing ferroxidase, plays a pivotal role in systemic copper and iron homeostasis. Its function as a potential spatial-molecular bridge linking copper and iron metabolism across zones is explicitly hypothesis-generating; direct evidence of zonal CP expression, activity gradients, and functional consequences for zonal ferroptosis/cuproptosis susceptibility is currently lacking and should be a priority for future zone-resolved studies. Its canonical function is to oxidize Fe^2+^ to Fe^3+^, facilitating iron binding to transferrin and thus regulating iron recycling and efflux [101]. We explicitly acknowledge that direct in vivo evidence demonstrating CP as a spatial-molecular bridge between these two pathways is currently lacking. However, we propose a forward-looking hypothesis that CP sits at a candidate regulatory crossroad. Furthermore, transcripts of CP exhibit a clear decreasing gradient along the portocentral axis in mouse liver, with highest expression in Zone 1 [96]. We believe retaining this streamlined section holds significant value for the field by highlighting a crucial knowledge gap and proposing a testable mechanism for future spatial biology research. Specifically, CP’s differential expression across the hepatic lobule could theoretically modulate the zonal thresholds for both cuproptosis and ferroptosis.

### 5.3. Dynamic Imbalance: From Homeostatic Gradients to Zonal Collapse

In the healthy liver, both iron and copper metabolism exhibit functional zonation, rather than steep absolute concentration gradients. It is hypothesized that Zone 3 has a microenvironment permissive to iron-dependent lipid peroxidation, while Zone 1 experiences higher copper flux due to the portal first-pass effect. This zonal homeostasis relies on the spatially coordinated expression of metal transporters (e.g., ferroportin 1, copper transporter 1, and ATP7B) and regulatory proteins (e.g., CP and ferritin). This physiological zonation maintains metal homeostasis. However, in diseased states, this gradient undergoes a dynamic imbalance. As a critical example, in hereditary hemochromatosis, systemic iron overload leads to extreme iron concentrations in portal blood, overriding typical physiological patterns and resulting in predominant Zone 1 iron deposition initially. In contrast, the microenvironmental vulnerability to ferroptosis we emphasize is heavily dependent on the specific disease etiology (such as steatosis patterns in MASLD or metabolic stress in alcoholic liver disease). This demonstrates that zonal metal distribution and subsequent cell death susceptibility are dynamically regulated by disease-specific contexts. For example, in MASLD, copper overload can disrupt lipid metabolism and insulin sensitivity, suggesting that copper dysregulation precedes overt tissue damage [13]. In patients with alcoholic hepatitis, M1 macrophage infiltration is positively correlated with ferroptosis and cuproptosis scores, indicating that the inflammatory microenvironment can simultaneously affect both metal pathways [102]. Research on engineered nanomaterials demonstrates that artificially inducing the intracellular co-accumulation of copper and iron can synergistically activate both death pathways [103] (note: this is an experimentally engineered co-toxicity model in a non-hepatic cancer setting, not evidence for physiological zonal co-collapse in liver disease). This finding provides a proof-of-concept for copper–iron synergistic toxicity but should not be extrapolated to physiological hepatic zonal pathology without direct liver-specific validation. Specifically, copper ions inhibit GPX4 and induce DLAT aggregation, whereas iron ions drive lipid peroxidation, collectively amplifying oxidative stress. This “metal co-toxicity effect”, if extrapolated to liver pathophysiology, would represent a zone-resolved co-collapse hypothesis that remains to be experimentally validated in hepatic models. Under pathological stress, it is hypothesized that Zone 1 may initiate cuproptosis, while Zone 3 may succumb to ferroptosis; however, this zonal collapse pattern is disease-specific and not a universal feature. This might compromise hepatic lobular structural integrity if it is validated. Therefore, although direct evidence is lacking, CP represents a potential spatial-molecular bridge connecting cuproptosis and ferroptosis, providing a theoretical foundation for understanding how zonal homeostasis collapses under pathological stress.

### 5.4. Disease Implications: Revisiting Liver Pathology

Traditional classifications of liver diseases are largely based on etiology (e.g., viral, alcohol, metabolic) or histological features (e.g., steatosis, fibrosis) and often overlook the spatial dimension of metalloptosis. Ferroptosis and cuproptosis are not isolated events but may cooperate in liver injury through shared regulatory nodes and hypothesized spatial complementarity, although direct coordinated execution remains unproven. For instance, excessive amounts of copper can directly bind to the cysteine 107 and cysteine 148 sites on GPX4, promoting its ubiquitination and degradation via the human T-cell leukemia virus type I binding protein 1-mediated autophagic pathway, thereby increasing sensitivity to ferroptosis [104]. Conversely, copper deficiency can also exacerbate ferroptosis by reducing GPX4 expression and impairing mitochondrial function [105]. This provides a rationale for combination targeted therapies. More importantly, diseases such as cholestasis, Wilson’s disease, and primary liver cancer all exhibit common features of copper–iron homeostasis imbalance [88,106,107], suggesting that the spatial redox–metalloptosis axis may be a possible integrative pathway that represents a shared downstream framework across multiple liver diseases.

Importantly, the zonal susceptibility to ferroptosis and cuproptosis is inherently dynamic and heavily context-dependent, rather than a universal, intrinsic property of any specific zone. Recognizing that the spatial redox–metalloptosis axis is heavily influenced by the specific etiology of the disease, we summarize the predominant zonal injury patterns and current evidence for metalloptosis across diverse pathological contexts (Table 2).

## 6. Therapeutic Prospects: From Zonal Blindness to Region-Aware Interventions

### 6.1. Analysis of “Zonal Blindness” in Current Therapies

In this section, we separate speculative research priorities (e.g., spatial cell-death risk scores, spatial drug targeting maps, labeled as “research concepts”) from approaches supported by preclinical proof-of-concept (e.g., zone-targeted nanoparticle delivery, labeled as “preclinical concepts”). Current therapeutic strategies for liver diseases generally lack consideration of hepatic spatial heterogeneity, resulting in significant “zonal blindness”. Most drugs targeting ferroptosis or cuproptosis were designed without distinguishing the differences in metabolic and redox microenvironments between Zone 3 and Zone 1. Sorafenib, a classic ferroptosis inducer in HCC treatment, can promote ferroptosis by inhibiting cystine uptake, but its selective effects on cells from different lobular zones remain unclear [106]. Although naturally active compounds such as artemisinin and curcumin have been shown to regulate ferroptosis via the NRF2 pathway and GPX4, their differential distribution between Zone 1 and Zone 3 is rarely considered [71]. The spatial distribution of nanomedicines (e.g., HKUST-1-based copper metal–organic frameworks, CaCuZC) capable of synergistically inducing ferroptosis and cuproptosis within the liver and their capacity for zone-specific accumulation have not been systematically evaluated [108,109]. Clinical trial results for the use of antioxidants in liver disease treatment are generally inconsistent, with significant heterogeneity in efficacy [110]. From the perspective of hepatic spatial heterogeneity, systemically administered drugs may theoretically face uneven regional distribution; however, this remains a hypothesis lacking direct pharmacokinetic evidence. Lower oxygen tension or distal sinusoidal location in Zone 3 does not necessarily imply inadequate drug exposure. Direct zone-resolved drug distribution studies are needed before this concern can be substantiated. This “zonal blindness” hypothesis represents a testable concept that warrants further investigation.

### 6.2. Charting a “Spatial Drug Targeting Map”

Emerging spatial multiomics technologies have revealed the spatiotemporal organization of the proteome, transcriptome, and metabolome within the liver at near-single-cell and subcellular resolution [111], providing the foundation for constructing a “spatial drug targeting map”. Major barriers to its realization include: (1) dynamic zonation (zonal boundaries shift with metabolic state and circadian rhythm); (2) disease-remodeled microcirculation (fibrosis and angiogenesis alter sinusoidal architecture); (3) cellular heterogeneity (non-parenchymal cells contribute to zonal signaling); (4) species differences (murine and human zonation differ significantly); and (5) technical limitations of current spatial profiling resolution. This map should integrate three key types of spatial information. Specifically, it should include the spatial distribution of iron and copper transporters (e.g., transferrin receptor 1, copper transporter 1, ATP7B) [87,112], as well as regional expression differences in key regulatory molecules (e.g., GPX4, ferroptosis suppressor protein 1, NRF2, FDX1) [106,113]. Additionally, it should incorporate gradient information on redox status and metal homeostasis. For example, Zone 3 is characterized by hypoxia and high CYP450 activity, making it prone to lipid peroxidation [30]. Thus, Zone 3 ferroptosis-related pathways represent potential therapeutic targets; however, we do not recommend specific modulators (e.g., ACSL4 inhibitors or GPX4 modulators) at this time, as zone-selective efficacy, delivery, and safety data are lacking. Future preclinical studies should first establish zone-resolved therapeutic indices before specific pharmacological recommendations can be made. In contrast, Zone 1 is characterized by active CP synthesis [96] and high copper flux due to the portal first-pass effect [27]. This zone may be more susceptible to cuproptosis inducers. Furthermore, RAB18 specifically regulates cuproptosis in HSCs, and allicin derivatives can selectively activate cuproptosis in HSCs without damaging hepatocytes [87], suggesting that the dual specificity of cell type and spatial location could serve as a key dimension of the map. In HCC, matrix stiffness regulates ferroptosis sensitivity via the Focal adhesion kinase-Yes-associated protein pathway [114], indicating that the physical microenvironment should also be incorporated into spatial targeting design. The CuFescore scoring system, constructed using The Cancer Genome Atlas and Gene Expression Omnibus databases, can predict patient cuproptosis/ferroptosis pathway activity and response to chemotherapy and immunotherapy [115]. Looking forward, integration with spatial transcriptomic data could enable the conceptualization of individualized zone-targeted strategies, although rigorous experimental validation is required before clinical application.

### 6.3. Zone-Targeted Strategies

Zone-targeted strategies based on spatial heterogeneity are emerging. On the one hand, nanocarrier technology can enable zone-specific delivery. For instance, copper-based nanomaterials that deliver cuprous ions via clustered regularly interspaced short palindromic repeats-associated protein 9 ribonucleoprotein complexes and inhibit ATPase copper transporting alpha expression can achieve copper accumulation within the tumor microenvironment, synergistically triggering cuproptosis and ferroptosis [116]. In another example, cascade nanoreactors such as CaCuZC utilize the tumor microenvironment to release Cu and Ca, inducing mitochondrial damage and enhancing immunogenic cell death [109]. On the other hand, molecular interventions can exploit zone-specific pathways. Pericentral ferroptosis resistance may be associated with the activation of the GRP78-PKA signaling axis. This axis exerts anti-ferroptotic effects through PKA-mediated phosphorylation of GRP75 at mitochondria-associated endoplasmic reticulum membranes, which stabilizes NRF2 and promotes antioxidant gene transcription [117]. In Zone 1, the modulation of CP (synthesized by hepatocytes and secreted into sinusoids) could regulate systemic and local copper homeostasis, influencing the cuproptosis threshold. With respect to liver fibrosis, the selective induction of ferroptosis or cuproptosis in HSCs has shown zonal protective potential. Specifically, ferroptosis can be induced by methyltransferase-like 9 knockdown, which downregulates SLC7A11 [69], or by the activation of autophagy via *N*^6^-methyladenosine (m^6^A) modification [118]. Similarly, cuproptosis can be induced by the induction of RAB18 phase separation by DATs [87]. In MASLD, managing copper levels may slow disease progression by improving lipid metabolism and insulin sensitivity [13], an effect that may primarily target periportal hepatocytes with active copper metabolism. Future studies should aim to validate the combination of spatially resolved imaging (e.g., ferroptosis visualization probes) [119] with zone-specific drug release systems to facilitate the eventual realization of “treatment by zone”.

### 6.4. Summary

Current therapeutic strategies for liver diseases generally overlook the spatial heterogeneity of ferroptosis and cuproptosis within the hepatic lobule, leading to limited efficacy and even contradictory results. Leveraging spatial multiomics and nanotechnology, charting a “spatial drug targeting map” is becoming feasible, with its core being the integration of multidimensional information, including metal distribution, redox status, key regulatory molecules, and the physical microenvironment. In this context, it is essential to distinguish established delivery technologies from speculative zone-specific applications. Nanocarrier-mediated precision delivery and cell type-specific death induction (e.g., HSC-targeted cuproptosis) represent established experimental platforms with proven efficacy in preclinical models. However, their precise application to exploit the hypothesized distinct vulnerabilities of Zone 3 and Zone 1 remains a future conceptual direction that requires rigorous validation through spatially resolved intervention studies. Therefore, future therapies should move beyond the “one-size-fits-all” model, with the long-term goal of translating these regional hypotheses into precise therapeutic levers once they are firmly validated.

## 7. Knowledge Gaps and Future Research Roadmap

### 7.1. Technological Gaps

Current research on the spatial heterogeneity of ferroptosis and cuproptosis in the liver is still limited by the lack of high-resolution, zone-specific detection and intervention tools. Although the near-infrared fluorescent probe TTM-4 enables near-infrared fluorescence visualization of lipid peroxidation during hepatocyte ferroptosis and has been successfully used for in vivo detection in murine MASLD, revealing correlations between CIDEC expression and ferroptosis marker signals [120], no comparable tools exist for real-time, in situ imaging of cuproptosis. This is particularly true for dynamically monitoring key events such as copper ion accumulation, mitochondrial lipoylated protein aggregation, or Fe-S cluster damage within different lobular zones (e.g., Zone 1 vs. Zone 3). Moreover, most current mechanistic studies rely on in vitro cells or bulk tissue homogenate analysis, making it difficult to decipher the differential death sensitivity of hepatocytes, HSCs, and nonparenchymal cells in the spatial dimension. For example, although RAB18-mediated cuproptosis in HSCs can be selectively activated by allicin derivatives [87], whether this process exhibits zonal preference remains unclear because of the lack of spatially resolved evidence. Future development is urgently needed for multimodal platforms to achieve precise mapping of the spatial redox–metalloptosis axis at the hepatic lobule scale. These platforms should integrate spatial transcriptomics, metal imaging (e.g., synchrotron X-ray fluorescence microscopy), redox state sensors, and zone-specific gene editing (e.g., adeno-associated virus serotype 8-mediated zonation-targeted Clustered Regularly Interspaced Short Palindromic Repeats).

### 7.2. Mechanistic Gaps

Although the roles of ferroptosis and cuproptosis in liver diseases have been widely reported, significant knowledge gaps remain regarding their interactive mechanisms within hepatic spatial zones. First, ferroptosis is characterized by iron-dependent lipid peroxidation, GPX4 inactivation, and GSH depletion, while cuproptosis is driven by copper binding to mitochondrial lipoylated proteins, leading to proteotoxic stress and metabolic collapse [121]. Despite extensive characterization of their core execution pathways, it remains unclear whether the molecular execution of these processes in Zone 3 versus Zone 1 involves zone-specific regulatory networks, given the involvement of nodes such as oxidative stress, mitochondrial dysfunction, and GSH-GPX4 pathway dysregulation [122]. For instance, 75 kDa glucose-regulated protein translocation to mitochondria-associated endoplasmic reticulum membranes and subsequent NRF2 activation under ferroptotic stress suggest that mitochondria-associated endoplasmic reticulum membrane dynamics may mark a ferroptosis-susceptible zone [117]. Yet, whether this mechanism is preferentially active in Zone 3 hepatocytes remains unclear. Second, as CP is a candidate link connecting copper and iron metabolism, direct evidence of how the expression gradient of CP across different liver zones influences local metal homeostasis and cell death thresholds is lacking. Furthermore, several studies have confirmed the synergistic interaction of ferroptosis and cuproptosis in HCC. For example, sorafenib enhances cuproptosis by inhibiting FDX1 degradation [106], and bimetallic nanoparticles simultaneously trigger both death modes [123]. Nonetheless, whether these interactions also occur in chronic liver diseases (e.g., MASLD, alcohol-associated liver disease, and fibrosis) remains unexplored. Additionally, whether they are modulated by zone-specific metabolic microenvironments (e.g., oxygen tension and substrate availability) is still unknown. Finally, the sensitivity of immune cells to ferroptosis or cuproptosis may influence intrahepatic inflammation and repair responses [23]. Still, no studies have investigated the differential responses of Kupffer cells, liver sinusoidal endothelial cells, or infiltrating lymphocytes to metalloptosis across different zones.

### 7.3. Translational Gaps

**Figure 4:**
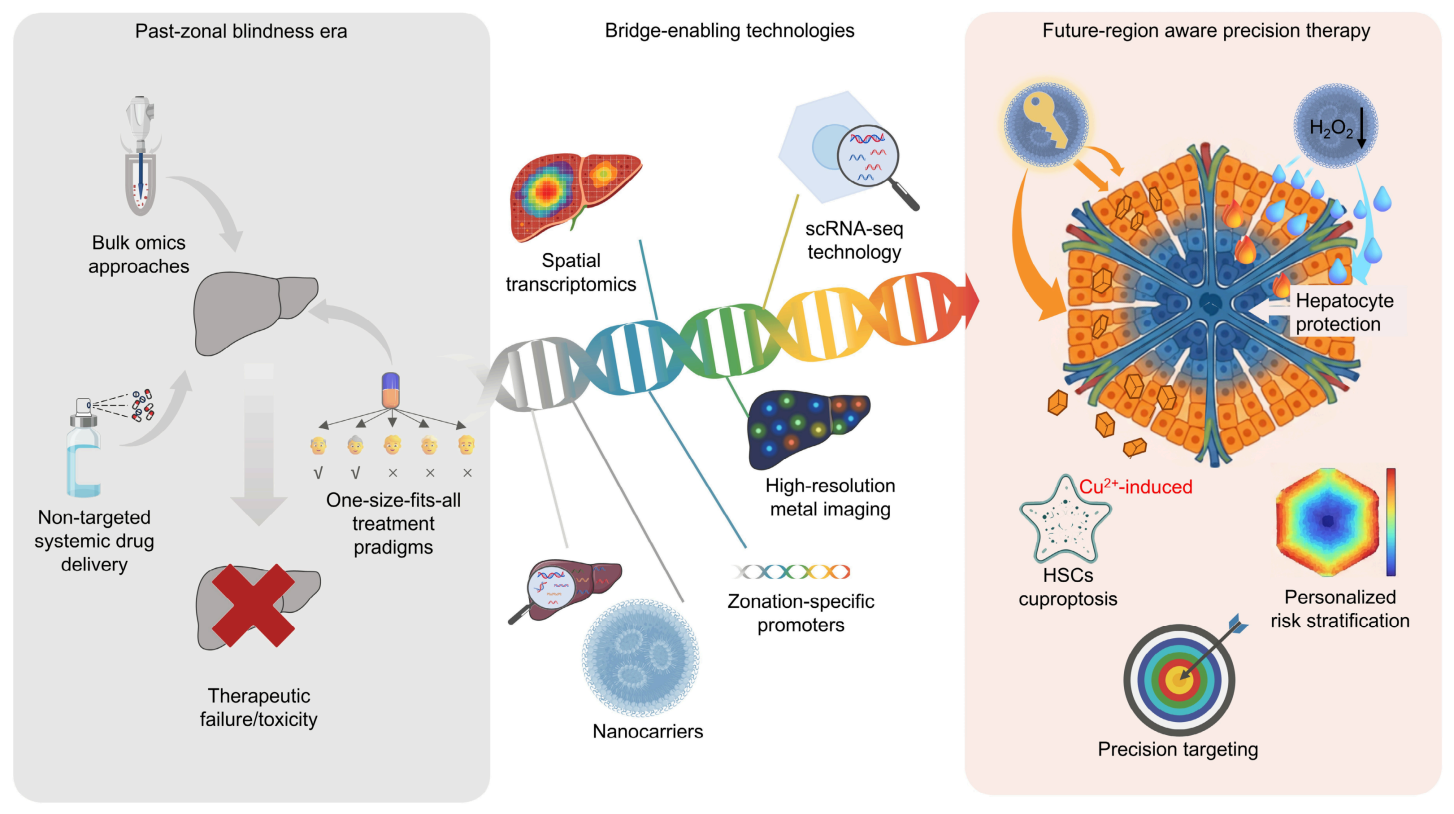
Proposed roadmap for spatial biology-based liver therapies. This figure illustrates the proposed transition from spatially agnostic treatment to spatially informed research and therapeutic development. The therapeutic roadmap depicted is conceptual and has not been clinically validated. Abbreviations: scRNA-seq, single-cell RNA sequencing; HSCs, hepatic stellate cells; H_2_O_2_, hydrogen peroxide.

A significant translational gap exists between mechanistic discoveries and clinical application. On one hand, ferroptosis inhibitors (e.g., UAMC-3203) can reduce steatosis and alanine aminotransferase elevation in MASLD mouse models [124]. Similarly, natural products ameliorate liver injury by modulating the NRF2, sirtuin, or p53 pathways [71], and targeting RAB18 to induce HSC cuproptosis shows anti-fibrotic potential [87]. However, most of these intervention strategies fail to account for hepatic spatial heterogeneity. This could lead to “one-size-fits-all” treatments that protect one zone while exacerbating injury in another. For example, systemic iron chelation could, in theory, alleviate iron-driven lipid peroxidation in Zone 1 (where pathological iron accumulates [10]) but might simultaneously impair iron-dependent essential functions in Zone 3 hepatocytes—a scenario that remains entirely hypothetical and is not supported by direct zone-resolved pharmacokinetic evidence. On the other hand, existing clinical biomarkers (e.g., serum iron, copper, oxidized phospholipids, 4-hydroxynonenal, and malondialdehyde), which reflect systemic metal imbalance and oxidative damage [122], cannot distinguish cell-death activity across lobular zones, limiting the precision of early diagnosis and therapeutic monitoring. Future translational research must focus on developing zone-aware delivery systems—such as nanocarriers driven by hepatocyte zonation-specific promoters or drug release platforms designed to respond to zonal metabolic features (e.g., Zone 3 hypoxia and high CYP450 activity)—as preclinical research directions; none of these delivery strategies has been validated clinically. Additionally, clinical cohorts incorporating spatial multiomics data from human liver tissues (e.g., spatial transcriptomics and metal distribution maps) should be established. These cohorts could, as a future research concept, be combined with imaging biomarkers to construct a conceptual spatial cell death risk score. We emphasize that this is a research concept only; it currently lacks derivation, weighting, validation, discrimination, calibration, and outcome associations, and is not proposed as a clinically usable tool. Ultimately, integrating regional vulnerability into therapeutic design is essential for truly advancing from “zonal blindness” to “region-aware” precision medicine for liver diseases (Figure 4). Looking ahead, AI-assisted analysis of spatial and single-cell omics may help prioritize zone-specific drug targets [125,126], although such computational predictions remain preclinical and require experimental validation.

## 8. Conclusions

In summary, this review proposes a testable spatial framework and research agenda rather than a validated map of zone-specific ferroptosis and cuproptosis. Ferroptosis and cuproptosis may exhibit mechanistic convergence through shared regulatory pathways, including GSH depletion, mitochondrial dysfunction, and oxidative stress, modulated by common regulators such as NRF2 and p53 [23]. The hypothesized spatial differences in ferroptosis and cuproptosis, if validated, would reveal a new dimension in the pathogenesis of liver diseases and open new avenues for diagnosis and treatment guided by regional vulnerability. Emerging nanoscale metal imaging technologies [18] and single-cell spatial multiomics analyses now provide the tools to rigorously test the hypothesis that Zone 3 is a ferroptosis-prone niche and Zone 1 is a cuproptosis-prone niche. While current evidence is largely inferential, validated human zonal biomarkers and zone-selective therapeutic strategies remain to be developed and are prerequisites for clinical translation. Acknowledging and dissecting the spatial logic of liver injury is therefore critical for transcending the traditional, spatially agnostic research paradigm in hepatology and moving beyond ‘zonal blindness’ toward spatially informed precision hepatology.

## Figures and Tables

**Table 1:** Summary of evidence for zonal susceptibility to ferroptosis and cuproptosis.

Zonal Claim/Disease Context	Zonal Pathology	Metal Distribution	Pathway Markers	Mechanistically Validated RCD Execution	Ref.
Hypothesized Zone 3 susceptibility to ferroptosis (MASLD/Metabolic injury)	Direct zonal pathology: Zone 3 steatosis; direct pathway-marker localization: lipid peroxidation/MDA adducts	Not tested	Inferred: Zone 3 lipogenic gene expression; upregulation of *ACSL4/GPX4* changes	Hypothetical/Not validated (Zone-specific ferroptosis rescue experiments are lacking)	[42,93,94]
Zone 1 iron deposition (Hereditary Hemochromatosis)	Direct zonal pathology: Zone 1 hepatocyte injury and periportal fibrosis	Direct zonal metal localization: Zone 1 iron accumulation (Perls’ stain)	Direct pathway-marker localization: Zone 1 MDA/4-HNE adducts; pathway activation: CREB-PC	Hypothetical/Not validated (Lipid peroxidation markers observed, but ferroptosis not validated via zonal rescue)	[10,95]
Zone 3 resistance to ferroptosis (Oncogenic context/HCC)	Direct zonal pathology: Zone 3 tumor origin; direct pathway-marker localization: GSTM2/3 expression	Not tested	Direct pathway-marker localization: GSTM2/3 mediated ferroptosis suppression; pathway activation: NRF2	Direct rescue-validated RCD modulation (non-zonal rescue; zone-specific origin and KO only): FSP1 overexpression on rescued GSTM3 deletion-induced tumorigenesis in bulk HDT/cell line models; zone-specificity from *Cyp1a2*-CreER lineage tracing and *Gstm3* KO	[73]
Hypothesized Zone 1 susceptibility to cuproptosis (Cholestatic disease/MASLD)	Direct zonal pathology: Zone 1 peribiliary fibrosis	Direct zonal metal localization: Zone 1 copper deposition (Dithiooxamide)	Inferred: FDX1, DLST downregulation; DLD/PDHB upregulation (Bioinformatics)	Hypothetical/Not validated (Cuproptosis validated in whole tissue/in vitro; no zone-specific rescue)	[85,88]
CP as a spatial link (Liver disease)	Not applicable	Not applicable	Direct pathway-marker localization: Zone 1 *CP* mRNA gradient (LCM & ISH)	Hypothetical/Not validated (mRNA gradients do not constitute RCD execution validation)	[96]

The Zonal Pathology, Metal Distribution, and Pathway Markers columns provide direct histological or molecular evidence associated with the respective zonal claims. The Mechanistically Validated RCD Execution column specifically states whether regulated cell death (ferroptosis or cuproptosis) has been directly executed and rescued within a specified lobular zone via pharmacological or genetic interventions. Most entries lack zone-specific rescue experiments; therefore, this column is labeled as “Hypothetical/Not validated” for the vast majority of entries, reflecting the honest hypothesis-driven nature of this review. The sole exception is the study (https://doi.org/10.1126/science.adv7129) [73], which directly validated zone-specific ferroptosis resistance via FSP1 rescue. Abbreviations: RCD, regulated cell death; Ref., reference; MASLD, metabolic dysfunction-associated steatotic liver disease; MDA, malondialdehyde; 4-HNE, 4-hydroxynonenal; CREB-PC, cAMP response element-binding protein pathway; HCC, hepatocellular carcinoma; GSTM, glutathione S-transferase Mu; NRF2, nuclear factor erythroid 2-related factor 2; KO, knockout; FSP1, ferroptosis suppressor protein 1; HDT, hydrodynamic transfection; Cyp1a2-CreER, cytochrome P450 1A2 promoter-driven Cre recombinase; FDX1, ferredoxin 1; DLST, dihydrolipoamide S-succinyltransferase; DLD, dihydrolipoamide dehydrogenase; PDHB, pyruvate dehydrogenase E1 subunit beta; CP, ceruloplasmin; LCM, laser capture microdissection; ISH, in situ hybridization. Methodological details (spatial method, disease stage, species/cell type, and mechanistic validation criteria) for each zonal claim are provided in Table S1.

**Table 2:** Disease-context framework for hepatic zonal injury and metalloptosis evidence.

Disease Context	Predominant Zonal Pathology	Hypothesized Zone 3 Ferroptosis Susceptibility Evidence	Hypothesized Zone 1 Cuproptosis Susceptibility Evidence	Disease Stage	Species/Cell Type	Spatial Resolution/Method	Refs.
MASLD	Adults: Zone 3 macrovesicular steatosis	Direct pathway-marker localization: Zone 3 MDA adducts (lipid peroxidation); Inferred: Zone 3 lipogenesis activation	Inferred: Upregulation of cuproptosis-related genes (*DLD*, *PDHB*) from Bulk RNA-seq	Steatosis/MASH/Fibrosis	Human & Murine/Hepatocytes	Histological zonation, IHC, Bulk transcriptomics	[42,43,85,93,94]
	Pediatric: Diffuse or Zone 1-predominant						
Alcohol-associated liver injury	Zone 3 (centrilobular) hepatocyte injury and steatosis	Direct pathway-marker localization: Zone 3 MDA/HNE adducts (alcoholic injury); Indirect, non-spatial molecular association: M1 macrophage–ferroptosis score correlations	Indirect, non-spatial molecular association: Positive correlations of M1 macrophage infiltration with cuproptosis score	Alcoholic hepatitis	Human/Macrophages & Hepatocytes	Bulk transcriptomics (in silico), Animal model	[95,102]
Drug-induced liver injury	Zone 3 (pericentral) injury for CYP-metabolized drugs	Inferred: TFEB-NCOA4-dependent ferritinophagy and ferroptosis pathway activation	No direct or relevant evidence identified	Acute toxicity	Murine/Hepatocytes & HepG2 cells (note: HepG2 is a non-spatial model)	Animal model, In vitro cell culture (non-spatial)	[75]
Hereditary iron overload	Early Stage: Zone 1 (periportal) iron deposition and fibrosis	Not supported for Zone 3: iron deposition is Zone 1 (periportal); MDA/4-HNE adducts predominate in Zone 1 (following iron deposition), not Zone 3; FGF21 rescue validated ferroptosis in whole-liver models but without zonal analysis	No direct or relevant evidence identified	Early to late fibrosis/cirrhosis	Human & Murine/Hepatocytes & Kupffer cells	Histology, IHC, Western blot, Bulk transcriptomics	[10,76,95]
Cholestatic disease	Zone 1 (periportal) and peribiliary injury; copper distribution is disease-dependent	No direct or relevant evidence identified	Hypothetical/Not validated (FDX1-mediated cuproptosis verified in whole tissue; Zone 1 copper accumulation observed but zonal execution not validated)	Cholestatic injury/MASH	Murine/Hepatocytes	BDL model, Histology, IHC, Western blot	[88]
HCC	Direct zonal pathology: Zone 3 (pericentral) preferential origin of oncogene-driven tumors	Direct rescue-validated RCD modulation (non-zonal rescue; zone-specific origin and KO only): Zone 3 Gstm2/3 expression resists ferroptosis, promoting tumor initiation (FSP1 rescue in bulk HDT/cell line models; zone-specificity from *Cyp1a2*-CreER lineage tracing and *Gstm3* KO)	Inferred: Bioinformatic signatures of cuproptosis-related genes predict poor prognosis and Sorafenib sensitivity	HCC/Cirrhosis	Murine & Human/Hepatocytes & HCC cells	Lineage tracing, Spatial transcriptomics, CRISPR/HDT, Bulk transcriptomics	[73,84,106]

Zonal susceptibility patterns are heavily context-dependent and may change during disease progression. “Direct zonal pathology” denotes definitive zonal injury; “Direct zonal metal localization” denotes spatially resolved metal deposition; “Direct pathway-marker localization” denotes spatially resolved pathway markers; “Direct rescue-validated RCD modulation” denotes rescue experiments confirming RCD execution; when rescue is performed in non-zonal models, this is explicitly noted in the cell. “Inferred” denotes conclusions drawn from histology or bioinformatics lacking zone-specific mechanistic validation; “Hypothetical/Not validated” denotes proposed mechanisms based on theoretical plausibility, not confirmed by zone-specific rescue experiments; “Indirect, non-spatial molecular association” refers to conclusions drawn solely from bulk transcriptomics or bioinformatics studies without spatial localization; “No direct or relevant evidence identified” is used to replace “N/A” and acknowledges that the biological process is applicable, but evidence is currently absent. HepG2 cells are explicitly annotated as a non-spatial model, indicating they lack hepatic zonation. Abbreviations: ref., reference; MASLD, metabolic dysfunction-associated steatotic liver disease; MDA, malondialdehyde; DLD, dihydrolipoamide dehydrogenase; PDHB, pyruvate dehydrogenase E1 subunit beta; RNA-seq, RNA sequencing; MASH, metabolic dysfunction-associated steatohepatitis; IHC, immunohistochemistry; HNE, 4-hydroxynonenal; CYP, cytochrome P450; TFEB, transcription factor EB; NCOA4, nuclear receptor coactivator 4; HepG2, human hepatoblastoma cell line; FGF21, fibroblast growth factor 21; FDX1, ferredoxin 1; BDL, bile duct ligation; HCC, hepatocellular carcinoma; RCD, regulated cell death; KO, knockout; Gstm, glutathione S-transferase Mu; FSP1, ferroptosis suppressor protein 1; HDT, hydrodynamic transfection; Cyp1a2, cytochrome P450 family 1 subfamily A member 2; CreER, Cre recombinase fused to estrogen receptor; CRISPR, clustered regularly interspersed short palindromic repeats.

## Data Availability

No new data were created or analyzed in this study. Data sharing is not applicable to this article.

## Supplementary Materials

The following supporting information can be downloaded at https://www.mdpi.com/article/10.3390/antiox15091053/s1, Table S1: Detailed spatial methodologies and mechanistic validation criteria for zonal metalloptosis susceptibility.

## Abbreviations

The following abbreviations are used in this manuscript:

ACSL4	Acyl-CoA synthetase long-chain family member 4
APAP	Acetaminophen
ATP7B	ATPase copper transporting beta
BD	Bile duct
CoQ10	Coenzyme Q10
CP	Ceruloplasmin
CPS1	Carbamoyl phosphate synthetase 1
CV	Central vein
CYP2E1	Cytochrome P450 family 2 subfamily E member 1
CYP450	Cytochrome P450
DHODH	Dihydroorotate dehydrogenase
DILI	Drug-induced liver injury
DLAT	Dihydrolipoamide S-acetyltransferase
ETC	Electron transport chain
FDX1	Ferredoxin 1
FTH1	Ferritin heavy chain 1
G6PC	Glucose-6-phosphatase catalytic
GCK	Glucokinase
GLUL	Glutamate-ammonia ligase
GPX4	Glutathione peroxidase 4
GSH	Glutathione
H_2_O_2_	Hydrogen peroxide
HA	Hepatic artery
HCC	Hepatocellular carcinoma
HSCs	Hepatic stellate cells
IHC	Immunohistochemistry
LIAS	Lipoic acid synthetase
MASLD	Metabolic dysfunction-associated steatotic liver disease
MASH	Metabolic dysfunction-associated steatohepatitis
MDA	Malondialdehyde
NCOA4	Nuclear receptor coactivator 4
NRF2	Nuclear factor erythroid 2-related factor 2
OTC	Ornithine transcarbamylase
PEPCK	Phosphoenolpyruvate carboxykinase
PKLR	Pyruvate kinase L/R
PV	Portal vein
RAB18	Ras-related protein Rab-18
RCD	Regulated cell death
ROS	Reactive oxygen species
scRNA-seq	Single-cell RNA sequencing
SLC7A11	Solute carrier family 7 member 11
STEAP	Six-transmembrane epithelial antigen of the prostate
TCA	Tricarboxylic acid
VLDL	Very low-density lipoprotein

## References

[1] Yang K., Kim K., Ryu T., Shim Y.R., Kim H.H., Choi S.E., Kim M.J., Chung K.P.S., Lee E., Lee K.W. (2025). Binge drinking triggers VGLUT3-mediated glutamate secretion and subsequent hepatic inflammation by activating mGluR5/NOX2 in Kupffer cells. Nat. Commun..

[2] Burghart L., Ferenci P., Petrenko O., Mandorfer M., Schwarz M., Gschwantler M., Trauner M., Reiberger T., Stattermayer A.F. (2024). Portal hypertension and its prognostic implications in patients with Wilson’s disease. Aliment. Pharmacol. Ther..

[3] Aizarani N., Saviano A., Sagar, Mailly L., Durand S., Herman J.S., Pessaux P., Baumert T.F., Grun D. (2019). A human liver cell atlas reveals heterogeneity and epithelial progenitors. Nature.

[4] Ben-Moshe S., Itzkovitz S. (2019). Spatial heterogeneity in the mammalian liver. Nat. Rev. Gastroenterol. Hepatol..

[5] Zhang L., Luo Y.L., Xiang Y., Bai X.Y., Qiang R.R., Zhang X., Yang Y.L., Liu X.L. (2024). Ferroptosis inhibitors: Past, present and future. Front. Pharmacol..

[6] Zizmare L., Hofmann U., Jarboui M.A., Klose F., Fraschka S., Matthes J., Kruger M., Schaeffeler E., Schwab M., Ueffing M. (2025). Cryogenic mouse tissue homogenization as an alternative to fresh-frozen biopsy use for genomics, transcriptomics, proteomics and metabolomics. Sci. Rep..

[7] Brockbals L., Ueland M., Fu S., Padula M.P. (2025). Development and thorough evaluation of a multi-omics sample preparation workflow for comprehensive LC-MS/MS-based metabolomics, lipidomics and proteomics datasets. Talanta.

[8] Santos A.A., Delgado T.C., Marques V., Ramirez-Moncayo C., Alonso C., Vidal-Puig A., Hall Z., Martinez-Chantar M.L., Rodrigues C.M.P. (2024). Spatial metabolomics and its application in the liver. Hepatology.

[9] Kurosaki S., Nakagawa H., Hayata Y., Kawamura S., Matsushita Y., Yamada T., Uchino K., Hayakawa Y., Suzuki N., Hata M. (2021). Cell fate analysis of zone 3 hepatocytes in liver injury and tumorigenesis. JHEP Rep..

[10] Yang Q., Wu Y., Liu W., Ou X., Zhang W., Wang J., Chang Y., Wang F., Gao M., Liu S. (2024). Zonated iron deposition in the periportal zone of the liver is associated with selectively enhanced lipid synthesis. Liver Int..

[11] Zhu W., Fu L., Cui Y., Tang Y., Liu K., Shi L., Gao Y., Li M., Huang L. (2025). Ferroptosis in liver fibrosis and its potential intervention strategy. Cell Biol. Toxicol..

[12] Chen X., Kang R., Kroemer G., Tang D. (2021). Broadening horizons: The role of ferroptosis in cancer. Nat. Rev. Clin. Oncol..

[13] Li Y., Qi P., Song S.Y., Wang Y., Wang H., Cao P., Liu Y., Wang Y. (2024). Elucidating cuproptosis in metabolic dysfunction-associated steatotic liver disease. Biomed. Pharmacother..

[14] Aigner E., Weiss G., Datz C. (2015). Dysregulation of iron and copper homeostasis in nonalcoholic fatty liver. World J. Hepatol..

[15] Krenkel O., Tacke F. (2017). Liver macrophages in tissue homeostasis and disease. Nat. Rev. Immunol..

[16] Lin P., Yan X., Jing S., Wu Y., Shan Y., Guo W., Gu J., Li Y., Zhang H., Li H. (2024). Single-cell and spatially resolved transcriptomics for liver biology. Hepatology.

[17] Barrows K.M., Porat-Shliom N. (2026). Hot zones for liver cancer: Metabolic zonation, ferroptosis, and the origins of HCC. Cancer Res..

[18] Zhang J., Dai W., Yang W., Luo W., Dong F., Hao J., Li R.Y., Xue C., Xie C., Sun L. (2025). Multimodal Profiling of Iron Heterogeneity at the Nanoscale. Nano Lett..

[19] Wang H., Wang H., Xiu T., Zhang X., Tang Y., Zhang W., Zhang W., Li P., Tang B. (2025). A “Cocktail” Fluorescent Probe for Multi-ROS Imaging Unveils Ferroptosis-Driven Liver Fibrosis Development. Angew. Chem. Int. Ed. Engl..

[20] Dong Z., Zhang Z., Peng Y., Chen Z., Lin L., Chen B., Zhao M., Gong M., Wang G., Aa J. (2026). Pien Tze Huang ameliorates alcohol-associated liver disease via suppressing oxidative stress and ferroptosis. Phytomedicine.

[21] Cho S.S., Yang J.H., Lee J.H., Baek J.S., Ku S.K., Cho I.J., Kim K.M., Ki S.H. (2022). Ferroptosis contribute to hepatic stellate cell activation and liver fibrogenesis. Free Radic. Biol. Med..

[22] Tan W., Zhang J., Chen L., Wang Y., Chen R., Zhang H., Liang F. (2024). Copper homeostasis and cuproptosis-related genes: Therapeutic perspectives in non-alcoholic fatty liver disease. Diabetes Obes. Metab..

[23] Wang L., Chen Liu Z., Wang D., Tang D. (2025). Cross-talks of GSH, mitochondria, RNA m6A modification, NRF2, and p53 between ferroptosis and cuproptosis in HCC: A review. Int. J. Biol. Macromol..

[24] Luan M., Zhu W., Feng Z., Jing F., Xing Y., Ma X., Wang Y., Ning B., Jia Y. (2026). MTF1 attenuates ferroptosis and cuproptosis synergistic potentiation in gastric cancer. Cell Death Differ..

[25] Liu N., Chen M. (2024). Crosstalk between ferroptosis and cuproptosis: From mechanism to potential clinical application. Biomed. Pharmacother..

[26] Ma C., Han L., Zhu Z., Heng Pang C., Pan G. (2022). Mineral metabolism and ferroptosis in non-alcoholic fatty liver diseases. Biochem. Pharmacol..

[27] van de Graaf S.F.J., Paulusma C.C., In Het Panhuis W. (2024). Getting in the zone: Metabolite transport across liver zones. Acta Physiol..

[28] Zhou Y., Zhao Y., Carbonaro M., Chen H., Germino M., Adler C., Ni M., Zhu Y.O., Kim S.Y., Altarejos J. (2024). Perturbed liver gene zonation in a mouse model of non-alcoholic steatohepatitis. Metabolism.

[29] Yasaka T.M., Kim C.K., Meadows V., Monga S.P. (2026). Zonation, Zonation, Zonation: The Real Estate of the Liver. Annu. Rev. Pathol..

[30] Jungermann K., Kietzmann T. (1996). Zonation of parenchymal and nonparenchymal metabolism in liver. Annu. Rev. Nutr..

[31] Jungermann K. (1986). Functional heterogeneity of periportal and perivenous hepatocytes. Enzyme.

[32] Bartl M., Pfaff M., Ghallab A., Driesch D., Henkel S.G., Hengstler J.G., Schuster S., Kaleta C., Gebhardt R., Zellmer S. (2015). Optimality in the zonation of ammonia detoxification in rodent liver. Arch. Toxicol..

[33] Albadry M., Kuttner J., Grzegorzewski J., Dirsch O., Kindler E., Klopfleisch R., Liska V., Moulisova V., Nickel S., Palek R. (2024). Cross-species variability in lobular geometry and cytochrome P450 hepatic zonation: Insights into CYP1A2, CYP2D6, CYP2E1 and CYP3A4. Front. Pharmacol..

[34] Kietzmann T. (2017). Metabolic zonation of the liver: The oxygen gradient revisited. Redox Biol..

[35] Kubota N., Kubota T., Kadowaki T. (2021). Midlobular zone 2 hepatocytes: A gatekeeper of liver homeostasis. Cell Metab..

[36] Nikopoulou C., Kleinenkuhnen N., Parekh S., Sandoval T., Ziegenhain C., Schneider F., Giavalisco P., Donahue K.F., Vesting A.J., Kirchner M. (2023). Spatial and single-cell profiling of the metabolome, transcriptome and epigenome of the aging mouse liver. Nat. Aging.

[37] Zhang T., Gu J., Wang X., Lu Y., Cai K., Li H., Nie Y., Chen X., Wang J. (2023). A novel liver zonation phenotype-associated molecular classification of hepatocellular carcinoma. Front. Immunol..

[38] Yu S., Wang H., Yang L., Yan Y., Cai Q., Ma D., Jiang L., Gao Z., Yu Z., Xia Z. (2022). Spatial transcriptome profiling of normal human liver. Sci. Data.

[39] Samarah L.Z., Zheng C., Xing X., Lee W.D., Afriat A., Chitra U., MacArthur M.R., Lu W., Jankowski C.S.R., Ma C. (2025). Spatial metabolic gradients in the liver and small intestine. Nature.

[40] Okada J., Landgraf A., Xiaoli A.M., Liu L., Horton M., Schuster V.L., Yang F., Sidoli S., Qiu Y., Kurland I.J. (2025). Spatial hepatocyte plasticity of gluconeogenesis during the metabolic transitions between fed, fasted and starvation states. Nat. Metab..

[41] Reza H.A., Santangelo C., Iwasawa K., Reza A.A., Sekiya S., Glaser K., Bondoc A., Merola J., Takebe T. (2025). Multi-zonal liver organoids from human pluripotent stem cells. Nature.

[42] Chalasani N., Wilson L., Kleiner D.E., Cummings O.W., Brunt E.M., Unalp A., Network N.C.R. (2008). Relationship of steatosis grade and zonal location to histological features of steatohepatitis in adult patients with non-alcoholic fatty liver disease. J. Hepatol..

[43] Carter-Kent C., Brunt E.M., Yerian L.M., Alkhouri N., Angulo P., Kohli R., Ling S.C., Xanthakos S.A., Whitington P.F., Charatcharoenwitthaya P. (2011). Relations of steatosis type, grade, and zonality to histological features in pediatric nonalcoholic fatty liver disease. J. Pediatr. Gastroenterol. Nutr..

[44] Lee-Montiel F.T., George S.M., Gough A.H., Sharma A.D., Wu J., DeBiasio R., Vernetti L.A., Taylor D.L. (2017). Control of oxygen tension recapitulates zone-specific functions in human liver microphysiology systems. Exp. Biol. Med..

[45] Savoca M., Takemoto K., Hu J., Li L., Jacob Kendrick B., Zhong Z., Lemasters J.J. (2024). MitoTracker Red for isolation of zone-specific hepatocytes and characterization of hepatic sublobular metabolism. Biochem. Biophys. Res. Commun..

[46] Penttila K.E. (1990). Role of cysteine and taurine in regulating glutathione synthesis by periportal and perivenous hepatocytes. Biochem. J..

[47] Weiss C.A.M., Brown L.A., Miranda L., Pellizzoni P., Steigerwald S., Remmert K., Hernandez J.M., Borgwardt K., Kleiner D.E., Ben-Moshe S. (2026). Single-cell spatial proteomics maps human liver zonation patterns and their vulnerability to disruption in tissue architecture. Nat. Metab..

[48] Ghafoory S., Stengl C., Kopany S., Mayadag M., Mechtel N., Murphy B., Schattschneider S., Wilhelmi N., Wolfl S. (2022). Oxygen Gradient Induced in Microfluidic Chips Can Be Used as a Model for Liver Zonation. Cells.

[49] Fuster-Martinez I., Bernal-Monterde V., Bidault G., Arbones-Mainar J.M., Vidal-Puig A. (2026). Hypoxia in MASLD: A spatial determinant of the pathogenesis. Trends Mol. Med..

[50] Vandenbon A., Mizuno R., Konishi R., Onishi M., Masuda K., Kobayashi Y., Kawamoto H., Suzuki A., He C., Nakamura Y. (2023). Murine breast cancers disorganize the liver transcriptome in a zonated manner. Commun. Biol..

[51] Klaren W.D., Vine D., Vogt S., Robertson L.W. (2018). Spatial distribution of metals within the liver acinus and their perturbation by PCB126. Env. Sci. Pollut. Res. Int..

[52] Doll S., Proneth B., Tyurina Y.Y., Panzilius E., Kobayashi S., Ingold I., Irmler M., Beckers J., Aichler M., Walch A. (2017). ACSL4 dictates ferroptosis sensitivity by shaping cellular lipid composition. Nat. Chem. Biol..

[53] Kim M.K., Paek K., Park K., Tak S., Na K., Choi J.H., Woo S.M., Kim H., Yoon Y.M., Chung S. (2025). A hepatic zonation chip with an oxygen concentration gradient embracing the spatial distribution of metabolic function. Biofabrication.

[54] Zhong G., Li L., Li Y., Ma F., Liao J., Li Y., Zhang H., Pan J., Hu L., Tang Z. (2023). Cuproptosis is involved in copper-induced hepatotoxicity in chickens. Sci. Total Env..

[55] Guilliams M., Bonnardel J., Haest B., Vanderborght B., Wagner C., Remmerie A., Bujko A., Martens L., Thone T., Browaeys R. (2022). Spatial proteogenomics reveals distinct and evolutionarily conserved hepatic macrophage niches. Cell.

[56] Ganz T. (2012). Macrophages and systemic iron homeostasis. J. Innate Immun..

[57] Harada M., Kumemura H., Sakisaka S., Shishido S., Taniguchi E., Kawaguchi T., Hanada S., Koga H., Kumashiro R., Ueno T. (2003). Wilson disease protein ATP7B is localized in the late endosomes in a polarized human hepatocyte cell line. Int. J. Mol. Med..

[58] Roelofsen H., Wolters H., Van Luyn M.J., Miura N., Kuipers F., Vonk R.J. (2000). Copper-induced apical trafficking of ATP7B in polarized hepatoma cells provides a mechanism for biliary copper excretion. Gastroenterology.

[59] Ziliotto N., Lencioni S., Cirinciani M., Zanardi A., Alessio M., Solda G., Da Pozzo E., Asselta R., Caricasole A. (2025). Functional characterisation of missense ceruloplasmin variants and real-world prevalence assessment of Aceruloplasminemia using population data. EBioMedicine.

[60] Li Z.D., Kang S., Li H., Yu P., Xie R., Li C., Jing Q., Gong Z., Li L., Li Z. (2025). Absence of astrocytic ceruloplasmin reverses the senescence process with aging of learning and memory abilities. Redox Biol..

[61] Jiang Q., Wang N., Lu S., Xiong J., Yuan Y., Liu J., Chen S. (2024). Targeting hepatic ceruloplasmin mitigates nonalcoholic steatohepatitis by modulating bile acid metabolism. J. Mol. Cell Biol..

[62] Wen H., Zi Y., Liu Z., Bai Y., Lin J., Wang H., Xu B., Wang J., Wang H. (2026). ACSL4-mediated astrocyte ferroptosis augments neuroinflammation and exacerbates NMOSD pathology. Cell Death Differ..

[63] Tu S., Zou Y., Yang M., Zhou X., Zheng X., Jiang Y., Wang H., Chen B., Qian Q., Dou X. (2025). Ferroptosis in hepatocellular carcinoma: Mechanisms and therapeutic implications. Biomed. Pharmacother..

[64] Liu J., Kang R., Tang D. (2022). Signaling pathways and defense mechanisms of ferroptosis. FEBS J..

[65] Cai X., Zhang Y., Qin W., Li X. (2026). Molecular code of ferroptosis: Emerging multi-dimensional modifications and therapeutic targets in hepatic disorders. Metabolism.

[66] Wu J., Wang Y., Jiang R., Xue R., Yin X., Wu M., Meng Q. (2021). Ferroptosis in liver disease: New insights into disease mechanisms. Cell Death Discov..

[67] Ji J., Jin Y., Ma S., Zhu Y., Bi X., You Q., Jiang Z. (2024). Discovery of a NCOA4 Degrader for Labile Iron-Dependent Ferroptosis Inhibition. J. Med. Chem..

[68] Yao F.F., Zhou S.Q., Zhang R.Z., Chen Y.N., Huang W., Yu K., Yang N.M., Qian X.J., Tie X.F., Xu J.L. (2024). CRISPR/Cas9 screen reveals that targeting TRIM34 enhances ferroptosis sensitivity and augments immunotherapy efficacy in hepatocellular carcinoma. Cancer Lett..

[69] Bi F.F., Qiu Y.X., Wu Z.F., Liu S.R., Zuo D.L., Huang Z.K., Li B.K., Yuan Y.F., Niu Y., Qiu J.L. (2023). METTL9-SLC7A11 axis promotes hepatocellular carcinoma progression through ferroptosis inhibition. Cell Death Discov..

[70] Feng Y., Shi M., Zhang Y., Li X., Yan L., Xu J., Liu C., Li M., Bai F., Yuan F. (2024). Protocatechuic acid relieves ferroptosis in hepatic lipotoxicity and steatosis via regulating NRF2 signaling pathway. Cell Biol. Toxicol..

[71] Liang Y., Qiu S., Zou Y., Luo L. (2024). Targeting ferroptosis with natural products in liver injury: New insights from molecular mechanisms to targeted therapies. Phytomedicine.

[72] Han J.F., Jia Z.Y., Fan X., Zhao X.Y., Cheng L.Y., Xia Y.X., Ji X.R., Zang W.Q. (2025). Mechanisms of ferroptosis in primary hepatocellular carcinoma and progress of artificial intelligence-based predictive modeling in hepatocellular carcinoma. World J. Gastroenterol..

[73] Guo J., Liang R., Chung A., Li Z., Li B., Chen E., Li L., Wang J., Hsieh M.H., Fang I.X. (2026). The origin of hepatocellular carcinoma depends on metabolic zonation. Science.

[74] Zeng X., Jiang W., Wu T., Li L., Fu F.D., Yao H., Wu D.B. (2025). Neonatal liver-derived FTH1-enriched extracellular vesicles attenuate ferroptosis and ameliorate MASLD pathogenesis. Free Radic. Biol. Med..

[75] Wang J., Song X., Hui Y., Dong B., Gong J., Zhao Y., Ji H., Qiu Y., Jiang S., Guo D. (2025). TFEB orchestrates ferritinophagy and ferroptosis in ionophore drug-induced hepatotoxicity: Unveiling a novel therapeutic avenue. Free Radic. Biol. Med..

[76] Wu A., Feng B., Yu J., Yan L., Che L., Zhuo Y., Luo Y., Yu B., Wu D., Chen D. (2021). Fibroblast growth factor 21 attenuates iron overload-induced liver injury and fibrosis by inhibiting ferroptosis. Redox Biol..

[77] Ang C.H., Arandjelovic P., Cheng J., Yang J., Guo F., Yu Y., Nelameham S., Whitehead L., Li J., Silver D.L. (2025). Self-maintenance of zonal hepatocytes during adult homeostasis and their complex plasticity upon distinct liver injuries. Cell Rep..

[78] Zhou H., Wang R., Zhu F., Lv J., Ren L., Yu B., Xiao J., Cheng Y., Wang H. (2026). Dynamically Reprograms Mitochondrial Respiration to Augment Cuproptosis in Cancer Therapy. Small.

[79] Tsvetkov P., Coy S., Petrova B., Dreishpoon M., Verma A., Abdusamad M., Rossen J., Joesch-Cohen L., Humeidi R., Spangler R.D. (2022). Copper induces cell death by targeting lipoylated TCA cycle proteins. Science.

[80] Xiong C., Ling H., Hao Q., Zhou X. (2023). Cuproptosis: P53-regulated metabolic cell death?. Cell Death Differ..

[81] Wang Y., Mang X., Guo X., Pu J. (2024). Distinct cuproptosis patterns in hepatocellular carcinoma patients correlate with unique immune microenvironment characteristics and cell-cell communication, contributing to varied overall survival outcomes. Front. Immunol..

[82] Yang Y., Wu J., Wang L., Ji G., Dang Y. (2024). Copper homeostasis and cuproptosis in health and disease. MedComm.

[83] Han X., Zhang X., Liu Z., Liu H., Wu D., He Y., Yuan K., Lyu Y., Liu X. (2025). Copper-Based Nanotubes That Enhance Starvation Therapy Through Cuproptosis for Synergistic Cancer Treatment. Adv. Sci..

[84] Nie G., Peng D., Wen N., Wang Y., Lu J., Li B. (2023). Cuproptosis-related genes score: A predictor for hepatocellular carcinoma prognosis, immunotherapy efficacy, and metabolic reprogramming. Front. Oncol..

[85] Wu C., Liu X., Zhong L., Zhou Y., Long L., Yi T., Chen S., Li Y., Chen Y., Shen L. (2023). Identification of Cuproptosis-Related Genes in Nonalcoholic Fatty Liver Disease. Oxid. Med. Cell Longev..

[86] Cai X., Deng J., Zhou X., Wang K., Cai H., Yan Y., Jiang J., Yang J., Gu J., Zhang Y. (2025). Comprehensive analysis of cuproptosis-related genes involved in immune infiltration and their use in the diagnosis of hepatic ischemia-reperfusion injury: An experimental study. Int. J. Surg..

[87] Tian H., Sun S., Qiu X., Wang J., Gao Y., Chen J., Han X., Bao Z., Guo X., Sun Y. (2025). Diallyl Trisulfide From Garlic Regulates RAB18 Phase Separation to Inhibit Lipophagy and Induce Cuproptosis in Hepatic Stellate Cells for Antifibrotic Effects. Adv. Sci..

[88] Guo Y.J., Yang M., Sun S.B., Zhong Z.H., Lu W.J., Zhang Z.N., Fan M.L., Zhang A.D., Zhang T.T., Wu Y. (2026). FDX1-mediated cuproptosis promotes cholestatic liver injury exacerbated by taurocholic acid-enhanced copper accumulation. Cell Death Discov..

[89] Fu Y., Hou L., Han K., Zhao C., Hu H., Yin S. (2025). The physiological role of copper: Dietary sources, metabolic regulation, and safety concerns. Clin. Nutr..

[90] Seubnooch P., Montani M., Dufour J.F., Masoodi M. (2024). Spatial lipidomics reveals zone-specific hepatic lipid alteration and remodeling in metabolic dysfunction-associated steatohepatitis. J. Lipid Res..

[91] Sun X., Wu J., Liu L., Chen Y., Tang Y., Liu S., Chen H., Jiang Y., Liu Y., Yuan H. (2022). Transcriptional switch of hepatocytes initiates macrophage recruitment and T-cell suppression in endotoxemia. J. Hepatol..

[92] Cheng R., Li Z., Luo W., Chen H., Deng T., Gong Z., Zheng Q., Li B., Zeng Y., Wang H. (2025). A Copper-Based Photothermal-Responsive Nanoplatform Reprograms Tumor Immunogenicity via Self-Amplified Cuproptosis for Synergistic Cancer Therapy. Adv. Sci..

[93] MacDonald G.A., Bridle K.R., Ward P.J., Walker N.I., Houglum K., George D.K., Smith J.L., Powell L.W., Crawford D.H., Ramm G.A. (2001). Lipid peroxidation in hepatic steatosis in humans is associated with hepatic fibrosis and occurs predominately in acinar zone 3. J. Gastroenterol. Hepatol..

[94] Paolini E., Longo M., Meroni M., Dongiovanni P. (2025). Hepatic Zonation in MASLD: Old Question, New Challenge in the Era of Spatial Omics. Int. J. Mol. Sci..

[95] Niemela O., Parkkila S., Britton R.S., Brunt E., Janney C., Bacon B. (1999). Hepatic lipid peroxidation in hereditary hemochromatosis and alcoholic liver injury. J. Lab. Clin. Med..

[96] Troadec M.B., Fautrel A., Drenou B., Leroyer P., Camberlein E., Turlin B., Guillouzo A., Brissot P., Loreal O. (2008). Transcripts of ceruloplasmin but not hepcidin, both major iron metabolism genes, exhibit a decreasing pattern along the portocentral axis of mouse liver. Biochim. Biophys. Acta Mol. Basis Dis..

[97] Liu T., Kong X., Qiao J., Wei J. (2025). Decoding Parkinson’s Disease: The interplay of cell death pathways, oxidative stress, and therapeutic innovations. Redox Biol..

[98] Mao C., Liu X.G., Zhang Y.L., Lei G., Yan Y.L., Lee H.M., Koppula P., Wu S.Q., Zhuang L., Fang B.L. (2021). DHODH-mediated ferroptosis defence is a targetable vulnerability in cancer. Nature.

[99] Yang W.S., SriRamaratnam R., Welsch M.E., Shimada K., Skouta R., Viswanathan V.S., Cheah J.H., Clemons P.A., Shamji A.F., Clish C.B. (2014). Regulation of ferroptotic cancer cell death by GPX4. Cell.

[100] Kera Y., Penttila K.E., Lindros K.O. (1988). Glutathione replenishment capacity is lower in isolated perivenous than in periportal hepatocytes. Biochem. J..

[101] Hellman N.E., Gitlin J.D. (2002). Ceruloplasmin metabolism and function. Annu. Rev. Nutr..

[102] Hou S., Wang D., Yuan X., Yuan X., Yuan Q. (2023). Identification of biomarkers co-associated with M1 macrophages, ferroptosis and cuproptosis in alcoholic hepatitis by bioinformatics and experimental verification. Front. Immunol..

[103] Liu M., Zheng J., Yu M., Wang Q., Yuan Y., Shao N., Yang X., Shen T., Wang L., Li A. (2026). Stimuli-Responsive CuFeTe(2) Nanosheets for Amplified Cuproptosis/Ferroptosis in Triple-Negative Breast Cancer Therapy. Adv. Sci..

[104] Xue Q., Yan D., Chen X., Li X., Kang R., Klionsky D.J., Kroemer G., Chen X., Tang D., Liu J. (2023). Copper-dependent autophagic degradation of GPX4 drives ferroptosis. Autophagy.

[105] Li F., Wu X., Liu H., Liu M., Yue Z., Wu Z., Liu L., Li F. (2022). Copper Depletion Strongly Enhances Ferroptosis via Mitochondrial Perturbation and Reduction in Antioxidative Mechanisms. Antioxidants.

[106] Wang W., Lu K., Jiang X., Wei Q., Zhu L., Wang X., Jin H., Feng L. (2023). Ferroptosis inducers enhanced cuproptosis induced by copper ionophores in primary liver cancer. J. Exp. Clin. Cancer Res..

[107] Sun X., Zhang X., Yan H., Wu H., Cao S., Zhao W., Dong T., Zhou A. (2023). Protective effect of curcumin on hepatolenticular degeneration through copper excretion and inhibition of ferroptosis. Phytomedicine.

[108] Tian H., Zhao S., Nice E.C., Huang C., He W., Zou B., Lin J. (2022). A cascaded copper-based nanocatalyst by modulating glutathione and cyclooxygenase-2 for hepatocellular carcinoma therapy. J. Colloid Interface Sci..

[109] Tang C., Liu K.R., Gao X.N., Kang H.M., Xie W.J., Chang J., Yin L.L., Kang J. (2025). A metal-organic framework functionalized CaO_2_-based cascade nanoreactor induces synergistic cuproptosis/ferroptosis and Ca^2+^ overload-mediated mitochondrial damage for enhanced sono-chemodynamic immunotherapy. Acta Biomater..

[110] Al-Madhagi H., Masoud A. (2024). Limitations and Challenges of Antioxidant Therapy. Phytother. Res..

[111] Suo Y., Thimme R., Bengsch B. (2026). Spatial single-cell omics: New insights into liver diseases. Gut.

[112] You H.M., Wang L., Bu F.T., Meng H.W., Huang C., Fang G.Y., Li J. (2022). Ferroptosis: Shedding Light on Mechanisms and Therapeutic Opportunities in Liver Diseases. Cells.

[113] He Y., Lin Y., Song J., Song M., Nie X., Sun H., Xu C., Han Z., Cai J. (2025). From mechanisms to medicine: Ferroptosis as a Therapeutic target in liver disorders. Cell Commun. Signal..

[114] Zhao Y., Ye Z., Liu Y., Zhang J., Kuermanbayi S., Zhou Y., Guo H., Xu F., Li F. (2024). Investigating the Role of Extracellular Matrix Stiffness in Modulating the Ferroptosis Process in Hepatocellular Carcinoma Cells via Scanning Electrochemical Microscopy. Anal. Chem..

[115] Shen Y., Li D., Liang Q., Yang M., Pan Y., Li H. (2022). Cross-talk between cuproptosis and ferroptosis regulators defines the tumor microenvironment for the prediction of prognosis and therapies in lung adenocarcinoma. Front. Immunol..

[116] Wu X., Bai Z., Wang H., Wang H., Hou D., Xu Y., Wo G., Cheng H., Sun D., Tao W. (2024). CRISPR-Cas9 gene editing strengthens cuproptosis/chemodynamic/ferroptosis synergistic cancer therapy. Acta Pharm. Sin. B.

[117] Liu H., Zheng S.L., Hou G.X., Dai J.R., Zhao Y.N., Yang F., Xiang Z.Y., Zhang W.X., Wang X.W., Gong Y.F. (2025). AKAP1/PKA-mediated GRP75 phosphorylation at mitochondria-associated endoplasmic reticulum membranes protects cancer cells against ferroptosis. Cell Death Differ..

[118] Shen M., Li Y.J., Wang Y.Q., Shao J.J., Zhang F., Yin G.P., Chen A.P., Zhang Z.L., Zheng S.Z. (2021). N^6^-methyladenosine modification regulates ferroptosis through autophagy signaling pathway in hepatic stellate cells. Redox Biol..

[119] Yin J., Xu L., Yang H., Qi W., Ren X., Zheng X., Shao X., Cheng T., Lin W. (2024). Construction of a Label-Detection Integrated Visual Probe to Reveal the Double-Edged Sword Principle of Ferroptosis in Liver Injury. Anal. Chem..

[120] Pham L.B.H., Kim T., Kim S., Kim Y.S., Kim J., Kim K., Lim H., Shim W.S., Kim B., Yoo S.Y. (2026). Novel near-infrared probe for monitoring lipid peroxidation-mediated viscosity change in ferroptotic hepatocytes. Clin. Mol. Hepatol..

[121] Song Q., Yang Y., Yang L. (2025). Navigating Transition Metal-Dependent Cell Death: Mechanisms, Crosstalk, and Future Directions. Adv. Sci..

[122] Li Pomi F., Di Leo G., Genovese S., Borgia F., Gangemi S. (2025). Redox Network Dysfunction: Integrating Ferroptosis and Cuproptosis Across Human Diseases. Antioxidants.

[123] Liu C., Guo L., Cheng Y., Gao J., Pan H., Zhu J., Li D., Jiao L., Fu C. (2025). A Mitochondria-Targeted Nanozyme Platform for Multi-Pathway Tumor Therapy via Ferroptosis and Cuproptosis Regulation. Adv. Sci..

[124] Peleman C., Hellemans S., Veeckmans G., Arras W., Zheng H., Koeken I., Van San E., Hassannia B., Walravens M., Kayirangwa E. (2024). Ferroptosis is a targetable detrimental factor in metabolic dysfunction-associated steatotic liver disease. Cell Death Differ..

[125] Guo C., Li Q., Liang W., Lu J., Yue W., Tian S., Zhang W. (2026). AI-enabled target discovery in traditional Chinese medicine: From computational prediction to experimental validation. Targetome.

[126] Qian J., Bao H., Yang H., Zhang J., Shao X., Fan X. (2026). AI-driven deciphering of cell niches with single-cell and spatial omics: A new perspective for traditional Chinese medicine research. Targetome.

