# Supplementary material for: The Spatial Redox–Metalloptosis Axis in Liver Disease: A Hypothesis on Regional Susceptibility to Ferroptosis and Cuproptosis
Source: Antioxidants (Basel). 2026 Aug 23;15(9):1053. doi: 10.3390/antiox15091053 (PMC13603676; doi:10.3390/antiox15091053)
Supplement: Supplementary file 1 [file antioxidants-15-01053-s001.zip › antioxidants-4406194-supplementary.pdf]

Table S1. Detailed spatial methodologies and mechanistic validation criteria for zonal metalloptosis susceptibility.

Zonal Claim/Disease Context	Spatial Method	Disease Stage	Species/Cell Type	Mechanistic Validation Criteria	Refs.
Hypothesized Zone 3 susceptibility to ferroptosis (MASLD/Metabolic injury)	Histological zonation (H&E, IHC), Spatial transcriptomics review	Steatosis/MASH	Human & Murine/Hepatocytes	Pathway activation only (Hypothetical/Not validated)	[40,92, 93]
Zone 1 iron deposition (Hereditary Hemochromatosis)	Histology, IHC, Bulk RNA-seq	Early to late iron overload	Human/Hepatocytes	Pathway activation only	[10,94]
Zone 3 resistance to ferroptosis (Oncogenic context/HCC)	Lineage tracing, Spatial transcriptomics, CRISPR/HDT	HCC	Murine/Hepatocytes & HCC cells	Rescue-validated (FSP1 overexpression)	[72]
Hypothesized Zone 1 susceptibility to cuproptosis (Cholestatic disease/MASLD)	BDL model, IHC, Western blot, Bulk RNA-seq	Cholestatic injury/Steatohepatitis	Murine/Hepatocytes	Pathway activation only (Hypothetical/Not validated)	[84,87]
CP as a spatial link (Liver disease)	Laser Capture Microdissection, In situ hybridization	Healthy/Pathological	Murine/Hepatocytes	Not applicable	[95]

The table details the spatial methodologies, disease stages, species/cell types, and mechanistic validation criteria supporting the specific zonal claims summarized in Table 1. In the Mechanistic Validation Criteria column, ‘Pathway activation only’ indicates that molecular markers associated with ferroptosis or cuproptosis were observed (e.g., lipid peroxidation, gene/protein changes) but lacked zonal-specific pharmacological or genetic rescue experiments. ‘Rescue-validated’ denotes experiments confirming RCD modulation in zone-specific contexts through specific interventions (e.g., FSP1 overexpression). ‘Not applicable’ is used when the entry represents a structural/molecular link without direct cell death assessment. Abbreviations: ref., reference; MASLD, metabolic dysfunction-associated steatotic liver disease; H&E, hematoxylin and eosin; IHC, immunohistochemistry; RNA-seq, RNA sequencing; HCC, hepatocellular carcinoma; CRISPR, clustered regularly interspersed short palindromic repeats; HDT, hydrodynamic transfection; FSP1, ferroptosis suppressor protein 1; BDL, bile duct ligation; CP, ceruloplasmin.
